# Improved Lipophilicity and Aqueous Solubility Prediction with Composite Graph Neural Networks

**DOI:** 10.3390/molecules26206185

**Published:** 2021-10-13

**Authors:** Oliver Wieder, Mélaine Kuenemann, Marcus Wieder, Thomas Seidel, Christophe Meyer, Sharon D. Bryant, Thierry Langer

**Affiliations:** 1 Department of Pharmaceutical Chemistry, University of Vienna, Althanstraße 14, A-1090 Vienna, Austria; marcus.wieder@univie.ac.at (M.W.); thomas.seidel@univie.ac.at (T.S.); thierry.langer@univie.ac.at (T.L.); 2 Servier Research Institute-CentEx Biotechnology, 125 Chemin de Ronde, 78290 Croissy-sur-Seine, France; melaine.kuenemann@servier.com (M.K.); christophe.meyer@servier.com (C.M.); 3 Inte:Ligand Software Entwicklungs und Consulting GmbH, 74B/11 Mariahilferstrasse, 1070 Vienna, Austria; bryant@inteligand.com

**Keywords:** AI deep-learning, neural-networks, graph neural-networks, cheminformatics, molecular property, machine-learning, computational chemistry, lipophilicity, solubility

## Abstract

The accurate prediction of molecular properties, such as lipophilicity and aqueous solubility, are of great importance and pose challenges in several stages of the drug discovery pipeline. Machine learning methods, such as graph-based neural networks (GNNs), have shown exceptionally good performance in predicting these properties. In this work, we introduce a novel GNN architecture, called directed edge graph isomorphism network (D-GIN). It is composed of two distinct sub-architectures (D-MPNN, GIN) and achieves an improvement in accuracy over its sub-architectures employing various learning, and featurization strategies. We argue that combining models with different key aspects help make graph neural networks deeper and simultaneously increase their predictive power. Furthermore, we address current limitations in assessment of deep-learning models, namely, comparison of single training run performance metrics, and offer a more robust solution.

## 1. Introduction

Oral bio-availability, drug uptake, and ADME-related properties of small molecules are key properties in pharmacokinetics. For drugs to reach their intended target, they need to pass through several barriers either by passive diffusion or carrier-mediated uptake typically mediated by lipophilicity and aqueous solubility. Compounds with poor solubility are unable to achieve that and, therefore, pose a higher risk in attrition and overall cost during development [1].

Methods based on deep-learning have proven successful in predicting molecular properties [2] and are becoming more and more a routine part of the modern computer-aided drug design toolbox for molecular design and med-chem decision support. Since molecules can be represented as graphs, an obvious approach is to employ a graph-based architecture for deep-learning, which leads to the utilization of graph-based neural networks (GNNs). These kinds of networks are capable of learning representations for a specific task in an automated way and, therefore, can eliminate the complicated feature engineering process where domain specialists have to select the list of descriptors themselves [3]. They became increasingly popular in the last few years [4,5,6] especially due to their success in chemical property prediction [5,7,8,9,10,11].

One of the first GNN models used for physicochemical property prediction was introduced by Micheli [12] in 2009. It predicted the boiling point of alkanes with a recursive architecture for structured data input and achieved an improved state-of-the-art performance. Lusci et al. [13] were the first to apply an undirected cyclic graph recurrent neural network on predicting aqueous solubility successfully. In the following years, several recurrent, spatial, and spectral graph-based neural networks were introduced [3,14,15,16]. One of them was the message passing framework, which was extended to include directed edges [3]. This network, called directed-edge message passing network (D-MPNN), is one of the most successful GNNs to predict chemical properties [1].

Despite the success, one important limitation with message passing networks is the graph isomorphism problem, meaning that they are unaware of the structural role of each node or edge [17]. Most standard GNNs, such as the D-MPNN, are incapable of distinguishing between different types of graph structures to determine whether they are topologically identical [18]. Compounds, such as naphthalene and 1,1-bi(cyclopentane), are perceived as the same structure by these networks. This can be problematic because they have vastly different chemical properties. To address this issue, graph isomophism networks (GIN), another group of GNNs, have recently received attention [18,19]. They are capable of distinguishing between these compounds by reformulating the message passing framework to incorporate the Weisfeiler–Lehman (WL) hierarchy. They try to be at least as expressive as the Weisfeiler–Lehman graph isomorphism test (WL-test) [20] and have shown good results in chemical property prediction [18,19] despite often falling short with respect to speed and accuracy to other frameworks, such as the D-MPNN [21]. Inspired by the key property of the GIN and the success of the D-MPNN framework, we combined the key characteristics of both architectures. By doing so we not only address the isomorphism problem but also incorporate one of the most successful and powerful GNN frameworks to improve lipophilicity and aqueous solubility prediction.

When comparing new machine learning architectures with previously published methods, the standard approach is to compare single performance metrics, such as root mean squared error (RMSE) values on a test set to show model performance [21,22]. This can lead to reproducibility issues as stochastic algorithms like neural networks can vary greatly in their prediction, even without changing their hyperparameters, simply by using different training, validation, test set splits or non-deterministic weight initializations [23,24]. One of the reasons for this is the complex landscape that optimizers have to navigate through in modern machine learning models. In real world applications these landscapes can have multiple local minima and it is especially hard for non-deterministic optimization algorithms like stochastic gradient descent to find the global minimum, therefore often retrieving different results when repeated [25]. This problem can be intensified by using small datasets with different random splits for training and evaluation. Such an approach can lead the optimization algorithm into different local minima and makes it almost impossible for the model to generalize [2]. It is, therefore, difficult to compare different deep-learning model architectures with each other even when using the same data [23]. Another challenge is especially prominent in the GNN domain, where the optimal features for node or edge representation are unknown. Deep-learning benchmark studies often use the same data but different representations for their input data which makes it difficult to make a fair comparison between the models [2,3].

To mitigate these problems, we use the exact same data split to train, evaluate, and test each of the used models with different node and edge features, as well as learning strategies to obtain an average performance independent of the used features and training approaches. Such a procedure is time consuming as multiple models have to be evaluated several times. Nevertheless, obtaining a better overview of the behaviour of GNNs under these different constraints will facilitate the understanding of these architectures and ultimately help advance GNNs beyond the current hype to more explainable and robust models.

**Figure 1:**
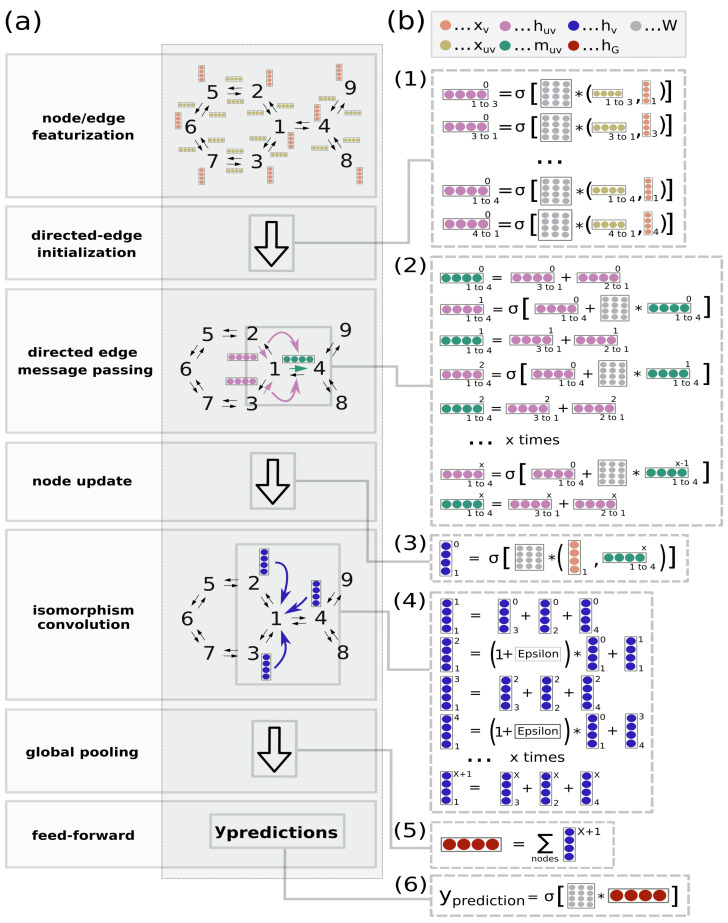
High level representation of the directed-edge graph isomorphism network (D-GIN) architecture for physicochemical prediction (logD, logS, or logP). (**a**) High level workflow depicting how a graph and its nodes and edges are featurized, then fed into the D-GIN to generate a molecular graph embedding. (**b**) The D-GIN architecture at a low level. Steps involved in generating input to make predictions: 1) Initial hidden directed-edge features ($h_{uv}^{0}$) are initialized by concatenating the corresponding node ($x_{v}$) and directed edge ($x_{uv}$) features. (2) Directed edge messages ($m_{uv}$) are used to update the hidden directed-edge features ($h_{uv}^{t}$). (3) Directed messages are combined with their corresponding hidden node features ($h_{v}$), and (4) iteratively updated by an additional trainable identifier (epsilon). (5) Hidden node features are aggregated to generate the molecular embedding ($h_{G}$) which is used as input for (6), the feed-forward neural network.

Our contribution is a novel graph neural network architecture called directed edge graph isomorphism network (D-GIN). It extends the directed edge message passing (D-MPNN) framework [1] by the graph isomorphism network (GIN) [18]. An overview of the D-GIN model is shown in Figure 1. Our novel architecture shows improved performance compared to its individual, less complex networks, and we demonstrate that combining models with different key aspects help make graph neural networks deeper while simultaneously increasing their predictive power. We evaluated our models by applying different learning and featurization strategies and compared their average performance under different constraints.

## 2. Materials and Methods

This section gives a detailed overview of the used data, molecular representation, and the different machine learning methods used throughout this work. The most common notations are shown in Table 1.

### 2.1. Experimental Data

A total of 10,617 molecules annotated with experimentally derived logD and logP values or logS and logP values were used for model training and predictions. The selected molecules were derived from the Delaney lipophilicity dataset containing experimentally evaluated logD and logP values at pH 7.4 [26] and an aqueous solubility set with logS and logP values [27]. Each dataset was evaluated and molecules were neutralized in both sets. For the aqueous solubility data, salts were stripped off and molecules with logS values lower than −10.0 or higher than 0.0 were removed. The original preprocessed and post-processed data can be found in the GitHub repository [28]. The splitting of each dataset into three subsets for training, evaluation, and testing was completed randomly in a ratio of 81:9:10 for the (training, evaluation, and testing). The data splitting was performed with the same seed for each of the models to be able to compare them using the exact same training, evaluation, and test data. The minimum value of each of the logD, logP, and logS properties was used as an offset to ensure only positive property values. The resulting lipophilicity dataset consisted of 4174 compounds. In total, 3380 were used for training, 376 for evaluating and model selection, and 418 for testing. The post processed solubility dataset contained 6443 molecules. Overall, 5219 compounds were allocated for training, 579 for evaluation, and model selection, and 645 for testing.

### 2.2. Training Approaches

The training strategies differ in the used dataset and the training target (logD, logP, or logS). Under these constraints, seven different types of strategies were used. The first multi-task learning strategy used a combined approach of logD, logP, and logS values referred to as “logD/P/S”. Three additional multi-task strategies utilized a combination of two physicochemical properties and are notated as either “logD/P”, “logD/S”, or “logS/P”. Three other single task strategies are only learned on a single physicochemical property and are referred to as either “single task logD”, “logP”, or “logS”. When physicochemical properties from different datasets were used, the individual datasets were first split into training, evaluation, and test sets. Afterwards, each physicochemical property was evaluated and tested individually so that the evaluation and test results of the multi-task learning approaches can be compared to those with a single-task learning strategy.

When testing either single-, or multi-task models, the combined root mean squared error (RMSE) for all properties was calculated as the measure for the best model. For logP, we only used the results from either the first multi-task approach (“multi-task logS/D/P”) or the single-task approach with logP values. The reasoning behind this was to use the same test and evaluation data for all models while trying to avoid an unbalanced data bias in favor of logP values. When training with two physicochemical properties where one was logP, we only used the data that had both properties. For example, when training on the lipophilicity dataset which had logP and logD values, we did not include logP compounds from the aqueous solubility dataset and vice versa.

### 2.3. Molecular Graphs

A graph is defined as G=(V,E), where *V* is a set of nodes and *E* denotes a set of edges. Let v∈V be a node with feature vector $x_{v}$ and $e{}_{uv}\in E$ be an edge pointing from *u* to *v* with feature vector $x^{e}{}_{uv}$. The adjacency matrix *A* shows the connectivity of the nodes and in our case it was binary as we did not weigh any connections. It is defined as a n×n matrix with $A{}_{uv}=1$ if $e{}_{uv}\in E$ and $A{}_{uv}=0$ if $e{}_{uv}\notin E$. We use directed, heterogeneous graphs where $e{}_{uv}\neq e{}_{v}u$. Heterogeneous graphs contain different types of nodes and edges with their corresponding featurizations.

### 2.4. Molecular Featurization

Five different types of edge and vertex featurizations *X* were being used for the GNNs. The detailed description of *x* and $x^{e}$ can be found in Table A1, Table A2, Table A3, Table A4, Table A5 and Table A6 in the Appendix A. The feature vectors for the non-GNN models consist of 8 different settings-fingerprints (ECFP or MACCSKeys-shown in Table A7 in the Appendix A) used either in combination with standardized RDKit [29] descriptors or without the descriptors. The descriptors were a combination of all possible and standardized RDKit descriptors, which had a total length of 208. The parametrization of the ECFP was either 1024, 1536, or 2048 bits with a radius of 4. Featurization 3 (Table A1 in the Appendix A) and 4 (Table A2 in the Appendix A) only differ in the way the size of ring systems are being represented. Either as a float value calculated by 1 divided by the size of the ring or as a one-hot encoding with 10 possibilities. The node and edge featurization in 5 (Table A3 in the Appendix A) includes two node features (chemical element and formal charge) and one edge feature (bond order). Featurization 6 (Table A4 in the Appendix A) includes the same node description as 5 and the edge featurization of 3. Featurization 7 (Table A5 in the Appendix A) has the same node featurization as 3 and the same edge featurization as 5. Featurization 8 (Table A6 in the Appendix A) includes a set of optimized node and edge features. This was performed by using a trained D-GIN model and then removing one node or edge feature at a time and observing the RMSE of the prediction. The five node features and the three edge features that had the biggest impact on the RMSE were then taken as the featurization. The graphs and their featurization were implemented using python version 3.7.8 and the toolkit CDPKit [30].

### 2.5. Directed-Edge GIN (D-GIN) and Reference Models

D-GIN is an extension of the directed-edge message passing neural network of Yang et al. [1] without the additional feature engineering in combination with the graph isomorphic network (GIN) of Xu et al. [18]. Its high level representation can be seen in Figure 1. The principle construction of the network can be seen in the Equations (1)–(8). First, the directed edges were initialized as

(1) $$h_{uw}^{0}=\tau \left(W_{init}\left(cat\left(x_{u},x^{e}{}_{uw}\right)\right)\right)$$

followed by a t∈1,…,T iteration of

(2) $$m_{uw}^{\left(t+1\right)}=\left\{\begin{array}{cc} \sum_{k\in N(u)/w}h_{ku}^{0}, & \mathrm{if}\;t==0.\\ \sum_{k\in N(u)/w}h_{ku}^{t}, & \mathrm{otherwise}. \end{array}\right.$$

(3) $$h_{uw}^{\left(t+1\right)}=\tau \left(h_{uw}^{0}+W_{m}m_{uw}^{t+1}\right)$$

after which the messages for each directed-edge was being summed as

(4) $$m_{u}=\sum_{w\in N(u)}h_{uw}^{T}$$

then the message $m_{u}$ was being concatenated as

(5) $$\begin{array}{c} h_{u}=\left\{\begin{array}{cc} cat(m_{u},x_{u}), & \mathrm{if}\;D-\mathrm{GIN}.\\ (W_{agg}\left(cat\left(m_{u},x_{u}\right)\right), & \mathrm{if}\;D-\mathrm{MPNN}. \end{array}\right. \end{array}$$

and another message passing over $l\in 1,\ldots ,T_{2}$ was performed by

(6) $$h_{u}^{\left(l\right)}=\left\{\begin{array}{cc} \sum_{w\in N(u)}h_{w}, & \mathrm{if}\;D-\mathrm{GIN}.\\ x_{u}, & \mathrm{if}\;\mathrm{GIN}. \end{array}\right.$$

(7) $$\begin{array}{cc} \; & h_{u}^{\left(l+1\right)}=\left(W_{agg}\left(1+\epsilon \right)h_{u}^{0}+h_{u}^{\left(l\right)}\right) \end{array}$$

afterwards the updated feature vectors $h_{node}^{T}$ of each node were aggregated over the whole molecule as

(8) $$\begin{array}{cc} \; & h_{G}=\sum_{h\in (H^{\left(T\right)})}h. \end{array}$$

The readout phase was then defined as $\hat{y}=f\left(h_{G}\right)$ where f(·) was a feed-forward neural network. The D-MPNN consisted of Equations (1)–(5) but then used the hidden feature vectors for each node directly by applying Equation (5) and then immediately Equation (7) to encode the whole graph as $h_{G}$.

GIN on the other hand was initialized and trained, as shown in Equation (6) in order to update the hidden feature vectors of each node. After l update step, the hidden feature vector of each node served as the input of Equation (7) to achieve the aggregated representation $h_{G}$ for the whole graph. D-GIN used all of these functions in a combined way described above Equations (1)–(8). The main principle behind this approach was to first use the key aspect of directed-edge message passing to propagate information via directed-edges to form messages Equations (1)–(4), which then updated the hidden node features Equations (5). These updated hidden node features were then used in the GIN message passing to further propagate information Equations (6) and (7) while also learning ϵ. These two information propagation phases are the key aspects of the two different sub-architectures.

### 2.6. Graph Neural Network Implementation, Training, and Hyper-Parameter Search

All GNNs have been implemented and trained using python version 3.7.8 and TensorFlow 2.3.0 [31]. We used TensorFlow’s keras models as our super-class and then transferred Equations (1)–(8) into the “fit” method of the keras model. A hyper-parameter search was conducted to find the best parameters which were further used to train all models. Further details on the hyper-parameters are given in the corresponding model’s configuration files accessible via the graph_networks Github repository [28].

Each GNN model type was trained twice with either 24 different settings when training on the logD or logS property or 12 on the logP property-in total 48 or 24 training runs per model type were performed. Each non-GNN model type was trained with 8 different settings. For training, evaluation, and testing we split each of the datasets as described in Section Experimental Data. Each of the GNNs were trained for 1600 epochs and the model with the best performance was identified using RMSE as the evaluation metric on the validation set. To evaluate the model type performance, we used the model with the best RMSE of the two runs performed for each model setting. When evaluating the average model type performance, the average RMSE of the different model settings was used for the calculation. To evaluate models with several properties, we summed all RMSEs. For example, when using logD and logP for training, we summed the RMSE of the logD and logP prediction on the evaluation set to receive a combined RMSE. When the combined RMSE was below the last best combined RMSE, the model weights were saved. We used these models to test the model on the test set. Each model was run two times and the results with the best test set performance were taken.

Additionally, the 95% confidence interval range was calculated by applying bootstrapping 100 times while leaving out 10% of the test dataset.

To generate consensus models between GNN and non-GNN models, we combined the best GNN model for each physicochemical property with the best non-GNN model. We did this by adding the predicted log values of one model with the other and then divided it by two. These hybrid models are then called according to their GNN model type plus consensus (e.g., D-GIN cons.).

### 2.7. Other Machine Learning Approaches

We used the random forest (RF), support vector machine (SVM), and k-nearest neighbor (K-NN) implementations of scikit-learn (Version 0.23.2 [32]). Default hyperparameters were used. The featurization is described in Table A7. When using descriptors as input, we standardized them with the scikit-learn StandardScaler. For the fingerprints and descriptors we used version 2020.09.2 of the RDKit [29] python package. Each of the models were trained in a single-task manner for each of the property values.

### 2.8. Hardware and Run-Time

Calculations were performed on machines within the Department of Pharmaceutical Sciences at the University of Vienna, Austria. We ran each model on a single CPU (Intel(R) Core(TM) i7-8700K CPU @ 3.70GHz). The run-time to fit the used RF, SVM, and KNN models with 3380 compounds on logD property values is approximately 50 s (RF), 25 s (SVM), and 0.5 s (KNN). When training the GNN model types on the 3380 logD compounds it takes for each epoch approximately 56 s (D-GIN), 35 s (D-MPNN), and 28 s (GIN).

## 3. Results and Discussion

For clarity, we define certain terms used throughout this publication that might have ambiguous meanings. The term “model type” refers to different kinds of machine learning algorithms. For example, a model type can be RF, SVM, KNN, D-GIN, GIN, or D-MPNN. The term “model” refers to a trained model instance with particular training and featurization strategies. The term “training strategy” is used to distinguish between different single- and multi-task training approaches trained with a combination of molecular properties. For example, logD/S/P is used to show that logD, logS, and logP values were used during training. The term “featurization strategy” is used to describe the different node and edge features utilized for the models to train on (Table A1, Table A2, Table A3, Table A4, Table A5 and Table A6 in the Appendix A). In addition, we distinguish between consensus (cons.) and non-consensus models. These hybrid models are a combination of the best GNN and best non-GNN models (SVM and D-GIN for logD and logS and RF and D-GIN for logP). To obtain consensus predictions, the predicted property values of the two models were combined and averaged. The averaged values were used as “new” predictions for the RMSE calculation and referred to their GNN model type plus cons (e.g., D-GIN cons).

Overall, 6 different machine learning model types were used in this study. The three GNN model types were D-MPNN, GIN, and D-GIN. The three non-GNN model types were random forest (RF) regression, support vector machines (SVM), and the k-nearest-neighbor (KNN) algorithm. Each model type was trained with the same hyperparameters, but 7 different learning strategies and 6 different node/edge featurization strategies. We trained each GNN model type for each physico-chemical property with all possible strategies twice. Subsequently, the best performing model from each of the two runs (measured on the evaluation set) was selected resulting in 24 models for the logD and logS property and 12 for the logP property, which were then used on the test set and their performance was reported.

The results of this approach are reported and discussed in two parts. First, we discuss different GNNs and non-GNN methods used in this work to identify the best performing model type according to its average performance across all used strategies (discussed in Section General Model Performance). Subsequently, we investigate the impact of the 6 different training strategies (i.e., multi-task vs. single task learning), as well as different featurizations on the performance (discussed in Sections Impact of Molecular Featurization and Impact of Training Strategies).

A dataset of 10,617 molecular structures with annotations for one of the three physico-chemical properties was assembled for model training, evaluation, and testing. It included 4174 logD, 6443 logS, and 10,617 logP experimentally measured values. The same training, evaluation, test set was used for all GNN and non-GNN model types.

### 3.1. General Model Performance

**Figure 2:**
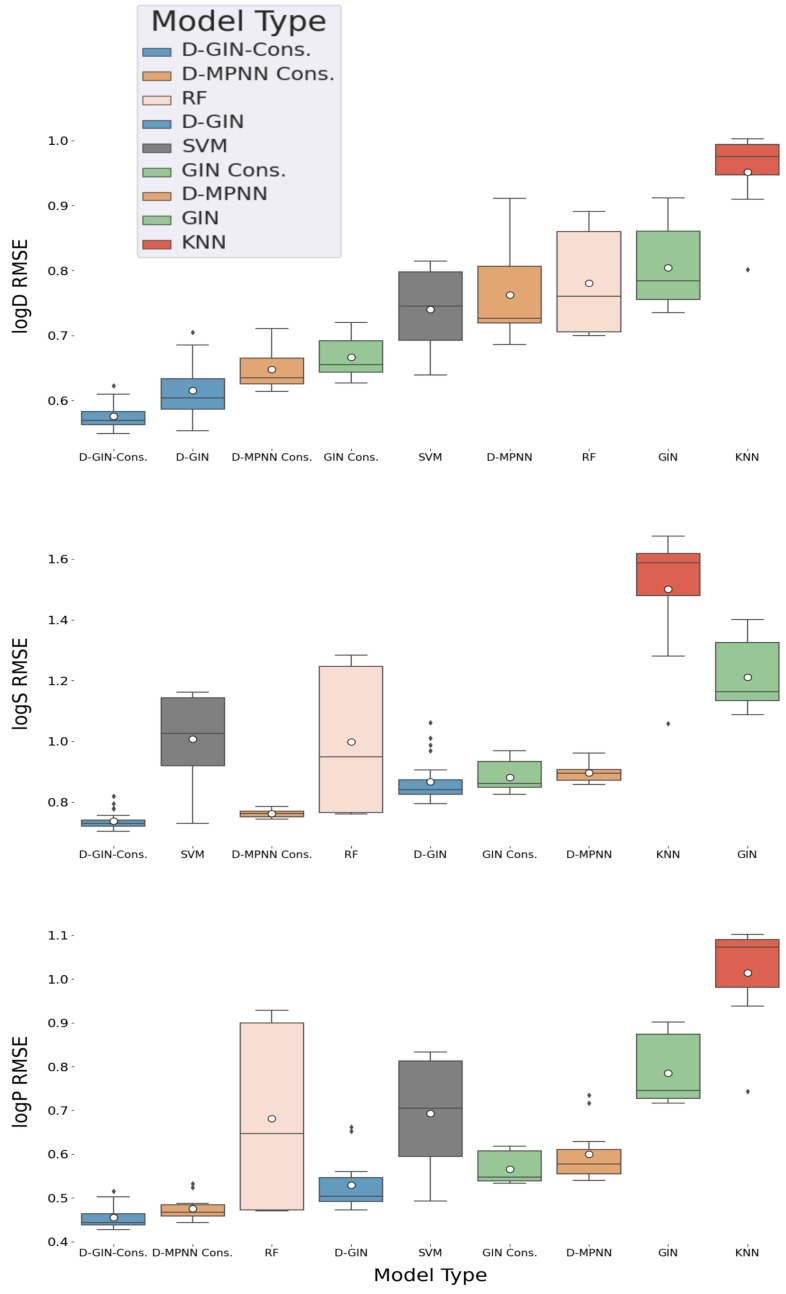
LogD, logS, and logP property prediction results for GNN and non-GNN model types with different featurization and training strategies. The different GNN architectures are colored in blue (D-GIN), orange (D MPNN), and green (GIN), the non-GNN architectures in gray (SVM), salmon pink (RF), and red (KNN) For logD and logS, 24 individual RMSE values were calculated for each model type. For logP 12 individual RMSE values were calculated. The individual boxplots show the average value of each model type as white dot and the median as a dark gray line. The values are listed in Table A8, Table A9, Table A10, Table A11, Table A12, Table A13, Table A14, Table A15, Table A16, Table A17, Table A18 and Table A19 in the Appendix A.

In the following, the reported results vary by the used model type. Each combination of featurization and training strategy was used to calculate a total of 24 RMSE values for the logD and logS property, and 12 for the logP property per model type. This resulted in a RMSE distribution shown in Table 2 and Figure 2. For each of these distributions, the average, minimum, and maximum RMSE was calculated and will be reported and discussed subsequently.

Table 2 shows the RMSE distribution average of the different machine learning model types regardless of their training and featurization strategy on the hold-out test set. For each value the standard error of the mean was calculated and added.

For logD property prediction, the D-GIN model type performed with mean, minimum, and maximum logD RMSE of 0.615 ± 0.039, 0.553, and 0.7048, and the corresponding consensus model with 0.575 ± 0.0192, 0.548, and 0.622, making it the best performing model type (results shown in Table 2, and Figure 2). The consensus GIN performed on average (distribution mean of logD RMSE values of 0.666 ± 0.029) better than the best non-GNN method (distribution mean logD RMSE of 0.740 ± 0.068).

For the logS prediction, the best model type was the D-GIN consensus model with a average RMSE value of 0.738 ± 0.028 (shown in Table 2 and Figure 2). It performed on average better than the best performing non-GNN model type (SVM), which performed with an average RMSE value of 1.006 ± 0.154 (but it also had a single run with a RMSE value of 0.729 making it the model type with the best single run performance and highlighting the importance of multiple repetitions for reporting model type performances). The consensus D-MPNN also outperformed the D-GIN.

The consensus D-GIN (average RMSE value of 0.455 ± 0.028) and consensus D-MPNN (average RMSE value of 0.475 ± 0.027) showed the best average performance for logP prediction (Table 2, and Figure 2). The RF and SVM model types also performed with low minimum RMSE values of 0.470 and 0.493, respectively. However, their average RMSE values (RF: 0.681 ± 0.224 and SVM: 0.693 ± 0.134) were higher than the D-GIN and D-MPNN model types.

Consensus models are often used in deep learning applications typically combining either different models that were trained on slightly different training data or multiple model architectures with different strengths and weaknesses. Nevertheless, further investigations are required to give a rationale of why in all our invested cases, the consensus models performed better than their individual counterparts. Furthermore, it should be noted that a direct comparison between the average performance of the GNNs and non-GNN models (RF, SVM, and KNN) can be difficult since the amount of information about a single molecule fed to each of the different model classes is quite different. For example, the non-GNN methods used a wide range of different descriptors and fingerprints shown in Table A7.

**Figure 3:**
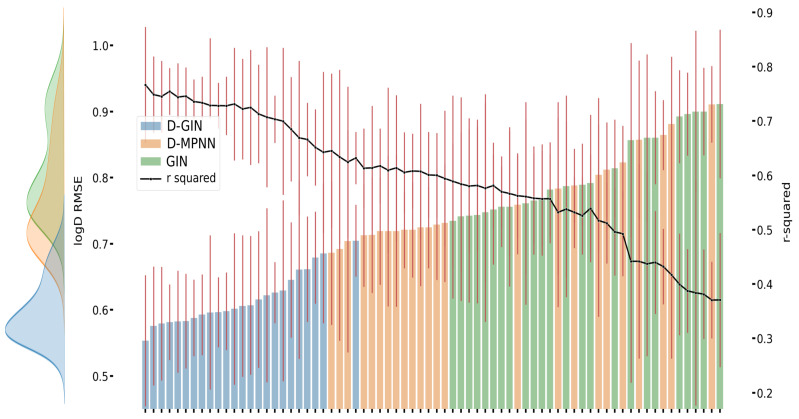
LogD prediction results for each GNN model instance. The left y-axis specifies the logD RMSE and the right, secondary x-axis the corresponding $r^{2}$ values for each GNN model. D-GIN is colored blue, D-MPNN orange, and GIN green. Each of the bars represent a different trained model-a detailed description can be found in Figure A1 in the Appendix A. The accumulated kernel density for each model type is shown on the very left side. The red lines correspond to the 95% confidence intervals. The model names are a combination of model type (D-GIN, GIN, D-MPNN), training approach and featurization type-a detailed description of each model name can be found in Table A8, Table A9, Table A10, Table A11, Table A12, Table A13, Table A14, Table A15, Table A16, Table A17, Table A18 and Table A19 in the Appendix A.

**Figure 4:**
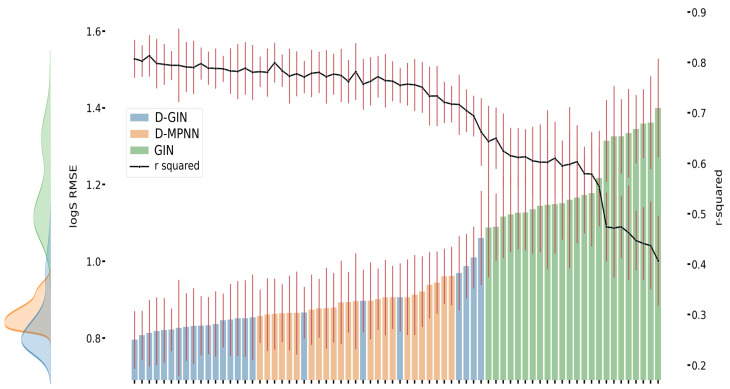
LogS prediction results for each GNN model instance. The left y-axis specifies the logS RMSE and the right, secondary x-axis the corresponding $r^{2}$ values for each GNN model. D-GIN is colored blue, D-MPNN orange, and GIN green. Each of the bars represent a different trained model-a detailed description can be found in Figure A3 in the Appendix A. The accumulated kernel density for each model type is shown on the very left side. The red lines correspond to the 95% confidence intervals. The model names are a combination of model type (D-GIN, GIN, D-MPNN), training approach and featurization type-a detailed description of each model name can be found in Table A8, Table A9, Table A10, Table A11, Table A12, Table A13, Table A14, Table A15, Table A16, Table A17, Table A18 and Table A19 in the Appendix A.

**Figure 5:**
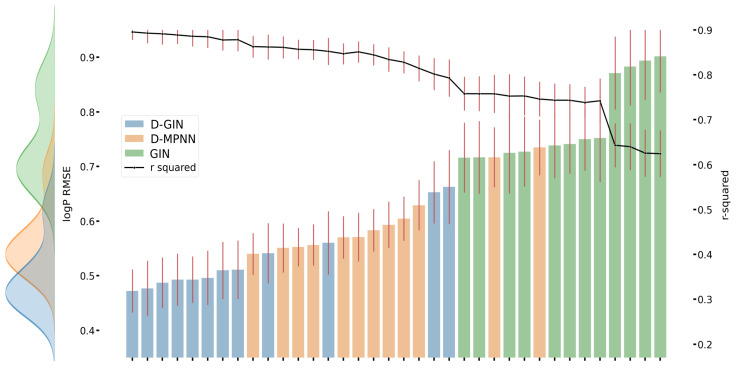
LogP prediction results for each GNN model instance. The left y-axis specifies the logP RMSE and the right, secondary x-axis the corresponding $r^{2}$ values for each GNN model. D-GIN is colored blue, D-MPNN orange, and GIN green. Each of the bars represent a different trained model-a detailed description can be found in Figure A2 in the Appendix A. The accumulated kernel density for each model type is shown on the very left side. The red lines correspond to the 95% confidence intervals. The model names are a combination of model type (D-GIN, GIN, D-MPNN), training approach and featurization type-a detailed description of each model name can be found in Table A8, Table A9, Table A10, Table A11, Table A12, Table A13, Table A14, Table A15, Table A16, Table A17, Table A18 and Table A19 in the Appendix A.

Figure 3, Figure 4 and Figure 5 show the best performing model architectures for prediction of each physicochemical property. Each plot shows the RMSE values for each GNN model applying all training and featurization strategies. It should be noted that the performance of many model types with different training or features do not significantly differ from each other and their CI overlap. Some trends are still visible: in Figure 3, Figure 4 and Figure 5, regardless of the physicochemical property, the D-GIN model type (shown in blue) performs overall better than the D-MPNN (shown in orange) or the GIN (shown in green).

The reason why the D-GIN outperforms the GIN and D-MPNN could be its higher complexity and network depth. It uses the key aspects of both sub-models and might be able to better abstract higher-order features. This could be facilitated by including skip connections between edge feature extraction mainly performed in the first (D-MPNN) and node feature extraction while learning ϵ in the second (GIN) part. This increased complexity could have helped to perform better than its individual parts.

### 3.2. Impact of Molecular Featurization

**Figure 6:**
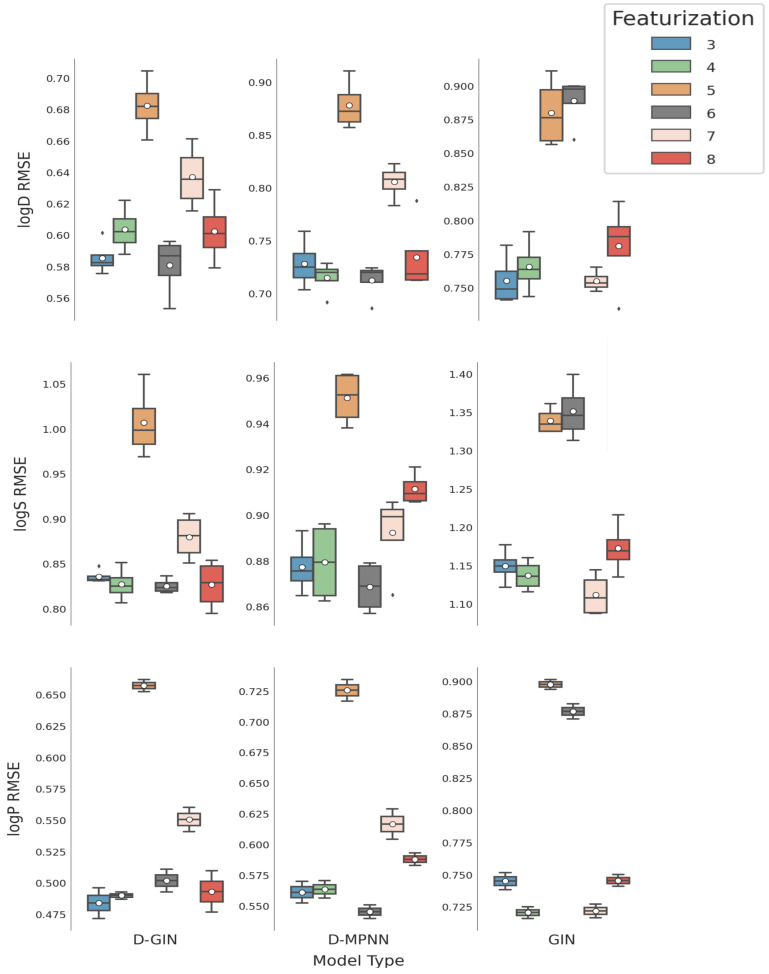
LogD, logS, and logP prediction results for all GNN model types depending on the featurization used (see Section 2.4 for a detailed description). The mean is shown as a white dot whereas the median is shown as a dark gray line. Exact values are listed in Table A8, Table A9, Table A10, Table A11, Table A12, Table A13, Table A14, Table A15, Table A16, Table A17, Table A18 and Table A19 in the Appendix A.

The average performance of each featurization strategy across all model types and training strategies is shown in Table 3. Considering the performance for all physicochemical properties, featurization strategy 5 showed the highest RMSE (mean logD/logS/logP RMSE of 0.813 ± 0.099, 1.099 ± 0.180, and 0.760 ± 0.110). This trend was also observed when separating according to the model type (shown in Table 4 and Figure 6). The reason for the relatively bad performance of featurization 5 might be that it only included two node properties (chemical element and formal charge), as well as only a single edge feature (bond order-Table A3 in the Appendix A).

Featurization 6 (Table A4 in the Appendix A) also displayed considerably worse performance than other strategies when used in combination with the GIN architecture, for which the mean RMSE performance for logD and logS properties were worse than using featurization strategy 5. One explanation could be that the GIN utilizes node features quite extensively and featurization 6 only included two node feature types similar to featurization 5. The additional edge features in strategy 6 without the appropriate architecture to deal with them could push the optimizer of the GIN network into the wrong direction rather than help with the property prediction.

Although it is easy to identify bad featurization strategies, it is difficult to come up with an unambiguous recommendation for the best performing featurization strategy. The mean RMSE across all training strategies and model types in Table 3 show that featurization 3 and 4 (Table A1 and Table A2 in the Appendix A) achieved very good results for logD with a RMSE value of 0.689 ± 0.079 and 0.694 ± 0.072, for logS with a RMSE 0.954 ± 0.146 and 0.948 ± 0.142 and for logP with a RMSE 0.596 ± 0.120 and 0.591 ± 0.105, respectively. Both featurization strategies utilize the maximum number of node and edge features used in this work. They only differ in the way molecular ring sizes are described. Featurization 3 used a float value calculated by 1 divided by the size of the ring system whereas featurization 4 used a one-hot encoding of ten instances (0,3,4,5,6,7,8,9,10,11).

Table 4 shows the mean RMSE values concerning featurization and model type. As performance criteria for featurization strategies we used the sum of model ranks in Table 4. Applying this approach, featurization 3 with two models as best and three models as second-best performers achieved a better ranking than featurization 4 with one model ranked best and two models as second best. Both strategies perform similarly well. Featurization 8 (shown in Table A6 in the Appendix A) used a set of optimized node and edge features. Node and edge features were optimized by masking single edge and node features at a time and evaluating their impact on the test set RMSE. The five node features and the three edge features that had the biggest impact on the RMSE were subsequently used. This approach also revealed that the size of ring systems for the node features appears to be of importance and was, therefore, included in 8. Using featurization 8, we were able to achieve two times the second-best performance. It shows an average good performance, but not as good as featurizations 3 or 4, even though its edge and node features were selected for maximum impact on the final prediction. The mean RMSE of featurization 6 and 7 (Table A5 in the Appendix A) in Table 3 show diminished results compared to featurization 3 and 4.

When evaluating the rank score, the featurization strategy that performs either best or second-best for each physicochemical property, the best featurization strategy was number 6. It was used in four of the best performing runs and once in a second-best run. However, it only performed well in combination with two GNN architectures (D-GIN and D-MPNN) and strongly underperformed with the GIN. The D-GIN and D-MPNN architecture types use primarily edge features for their information propagation and featurization strategy 6 provided these. It utilized only two-node feature types, potentially reducing the noise for the feature extraction to a minimum in this setting.

On average, featurization strategies 6 and 7 performed similarly well. However, when separating the results at a model type level, it became evident that there was a strong model architecture dependency, so it seems important to choose the features according to the architecture at hand (Figure 3, Figure 4 and Figure 5). Furthermore, featurization 3 might perform worse than featurization 6 or 7. Nevertheless, when unsure which features to use, simply adding more features could be the safer option rather than using less. This observation is also supported by comparing featurization 3 or 4 to, e.g., 6, 7, or 8.

When analyzing the results for the non-GNN models and their different featurizations, the mean RMSE variance was large in comparison to the GNN models. Moreover, in similar deep-learning benchmark studies that predicted molecular properties, predominantly fingerprints have been used. From Table A13, Table A14 and Table A15 in the Appendix A, one can see that especially featurizations that include descriptors in addition to fingerprints perform exceptionally well. We think that when comparing GNN with non-GNN models, differences in used features should be taken into consideration when trying to identify and understand (deep-learning) method performance.

### 3.3. Impact of Training Strategies

The impact of different training strategies are shown in Table 3. The lowest mean logD RMSE can be obtained by a multi-task strategy that involves learning on both logD and logP values. This is similar to the best training strategy for the logS property, which is a multi-task approach including logS and logP properties. As for the logP property, the best approach is a single-task strategy including logP values, however the multi-task approach which combines all physicochemical properties achieves similarly good performance.

**Figure 7:**
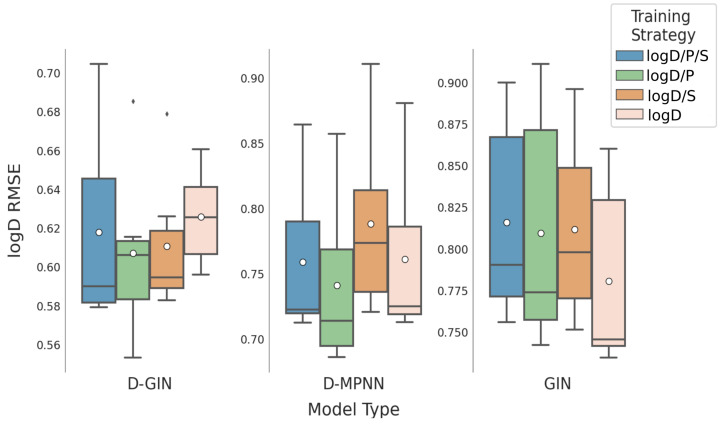
LogD prediction results for all GNN model types according to the used training strategy. Blue box shows the performance of the multi-task training strategy using logD, logS, and logP. The green and orange box show the results utilizing a combination of logD and logP and logD and logS for training. The salmon pink box shows the results using logD for training. The mean is shown as a white dot whereas the median is shown as a dark gray line. Exact values are listed in Table A8, Table A9, Table A10 and Table A11 in the Appendix A.

**Figure 8:**
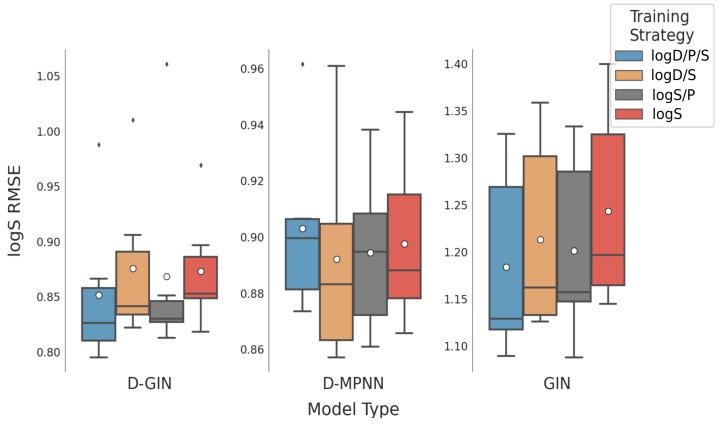
LogS prediction results for all GNN model types according to the used training strategy. The blue box shows the performance of the multi-task training strategy using logD, logS, and logP. The gray and orange box show the results utilizing a combination of logS and logP and logD and logS properties, respectively. The red box shows the results using logS for training. The mean is shown as a white dot whereas the median is shown as a dark gray line. Exact values are listed in Table A12, Table A13, Table A14 and Table A15 in the Appendix A.

**Figure 9:**
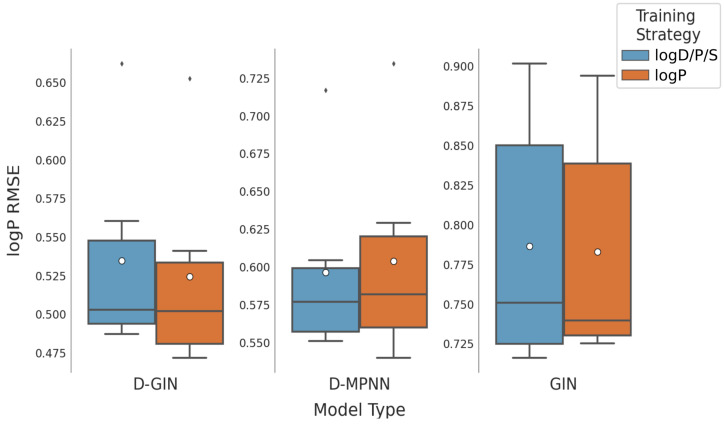
LogP prediction results for all GNN model types according to the used training strategy. Blue box shows the performance of the multi-task training strategy using logD, logS, and logP. The dark orange box shows the results using logP for training. The mean is shown as a white dot, whereas the median is shown as a dark gray line. Exact values are listed in Table A16, Table A17, Table A18 and Table A19 in the Appendix A.

When analyzing the logD/S/P RMSE predictions with respect to training strategy and model type, Table 5 and Figure 7, Figure 8 and Figure 9 show that there is no particularly favorable learning strategy for any of the model types. The datasets used in this study are specific for one particular physicochemical property. When comparing different learning strategies we thus focused on one particular physicochemical property for each model type. Starting with the results for the prediction of the logD property in Table 5, we can see that the overall best model (red asterisk), as well as the two best models for each model type (dark gray), are multi-task models. In particular, the models with a combination of logD and logP properties perform well.

Considering all combinations of training and featurizations strategies for each model, the learning strategy with the best average, as well as the best minimum logD RMSE was obtained using the logD/P multi-task training approach resulting in RMSE values of 0.719 ± 0.105 and 0.553, respectively (Table 3. Yet, using this multi-task learning strategy we also obtained single run performance worse than using a single-task learning strategy with only logD values, showcasing once more the importance of validating multiple learning and featurization strategies. The results are similar for the prediction of logS values: again, the multi-task learning strategy performs better than its single task counterpart. The best model for logS prediction was obtained by training on logD, logS, and logP values. Considering all combinations of training and featurization strategies for each model, the best average, minimum, and maximum logS RMSE of 0.979 ± 0.166, 0.795, and 1.325, respectively, was observed during the multi-task training with all properties. We should note here that while it seems that the average performance is improved by multi-task learning, the variance of model performance is also increased.

## 4. Conclusions

We introduced the directed-edge graph isomorphism network (D-GIN), a novel graph neural network that showed improved average performance for aqueous solubility and lipophilicity prediction compared with other baseline models. We showed that by combining different models with distinct key characteristics, we can increase the depth of the model while also improving its predictive power. Furthermore, applying different training strategies and featurizations constraints enables to obtain more information regarding general, average model performance. This strategy showed that the D-GIN model outperforms other machine-learning models on average and argued that comparing the mean performance rather than single metric values of the best performing model type gives more insight into the general behavior and ultimately facilitates a better understanding and higher robustness of deep-learning models.

In concurrence with previous publications [33,34,35], we showed that there is a tendency towards multi-task learning approaches for the GNNs utilized in this survey. On average they performed better than their single-task counterpart for the corresponding physicochemical property. We could not find clear evidence that more than two properties increase the model’s performance.

Furthermore, we highlighted that the usage of additional features did not improve the GNN model performance. However, we also conclude that very little featurization led to the worst performance. In general it is necessary to be aware of the type of GNN that is used and whether its architecture focuses more on edge or node features. When trying to obtain the best performing model it can be advisable to do feature engineering, but when in doubt which features to use, it can be safer to use more than less. We showed that this awareness can help improve the GNNs predictive power at hand.

For the non-GNN models, we could conclude that by excessively adding descriptors to the molecular fingerprint the performance of these models increases substantially. We further argued that for future comparisons it would be advisable to include not only fingerprints but also descriptors to the non-GNN baseline models to be more competitive.

By combining the best GNN model with the best non-GNN model we could see a slight improvement in the overall performance in all cases. Consensus models have often shown to improve performance. However, in this case, further investigations are needed to attain to a conclusion on why this is the case.

We showed that advanced deep-learning methods such as GNNs do have great potential in the physicochemical property prediction area and, when applied properly, can serve as a promising and robust method for any computer-aided drug discovery pipeline, especially for chemical property prediction.

## Figures and Tables

**Table 1:** Common notations used throughout this publication.

Notation	Definition
τ	A non-linear function (e.g., sigmoid or relu)
cat(,)	Vector concatenation
*t*	Iterator of t steps
*G*	A graph
*V*	Set of nodes
*E*	Set of edges
*v*	Node *v* ∈ *V*
$e_{uv}$	Edge $e_{uv}$ ∈ *E* between node *u* and *v*
N(u)	Neighbors of node *u*
N(u)/w	Neighbors of node *u* except *w*
*n*	The number of nodes
*m*	The number of edges
*d*	The dimension of a node feature vector
*b*	The dimension of a edge feature vector
*X*∈${}^{n\times d}$	Feature matrix of a graph
*x${}_{v}$* $\in^{d}$	Feature vector of node *v*
$x^{e}{}_{uv}$∈${}^{b}$	Feature vector of edge $e_{uv}$
*h${}_{v}$* $\in^{c}$	Hidden feature vector of node *v*
*m${}_{v}$* $\in^{c}$	Message feature vector to node *v*
*h${}_{G}$* $\in^{c}$	Feature vector of the graph *G*
*h${}_{uv}$* $\in^{d}$	Hidden feature vector of edge $e_{uv}$
*m${}_{uv}$* $\in^{d}$	Message feature vector to edge $e_{uv}$
*W*	Weight matrix of a neural network
*A* $\in {\left\{1,0\right\}}^{\left|n|x|n\right|}$	Adjacency matrix
*RMSE*	Root mean squared error
*GNN*	Graph neural network
*GIN*	Graph isomorphic network as in [18]
ϵ	Epsilon as described in [18]
*D-MPNN*	Directed-edge message passing network as in [1]
*D-GIN*	Directed-edge graph isomorphic network
*CI*	95% confidence interval calculated via bootstrapping
f(·)	Feed forward neural network

**Table 2:** Overview of the best performing machine learning model types independent of training and featurization strategy for prediction of logD, logS, and logP. The performance was calculated as the distribution average over all used model root mean squared error (RMSE) values. In total 24 models were used for the loD and logS property, and 12 for the logP property. RMSE values highlighted in dark and light gray show the best and next best models. Red asterisks mark the lowest RMSE for the non-consensus models for each property prediction.

Molecular	Model Type	Mean	Min	Max
Property		RMSE	RMSE	RMSE
logD	D-GIN	0.615 ± 0.039 *	0.553	0.704
	D-MPNN	0.762 ± 0.065	0.686	0.911
	GIN	0.804 ± 0.061	0.738	0.911
	RF	0.780 ± 0.084	0.699	0.890
	SVM	0.740 ± 0.068	0.639	0.814
	KNN	0.951 ± 0.067	0.801	1.003
	D-GIN cons.	0.575 ± 0.019	0.548	0.622
	D-MPNN cons.	0.647 ± 0.028	0.613	0.710
	GIN cons.	0.666 ± 0.029	0.627	0.719
logS	D-GIN	0.867 ± 0.070 *	0.795	1.061
	D-MPNN	0.896 ± 0.030	0.857	0.961
	GIN	1.210 ± 0.102	1.088	1.400
	RF	0.997 ± 0.253	0.760	1.284
	SVM	1.006 ± 0.154	0.729	1.162
	KNN	1.500 ± 0.217	1.057	1.676
	D-GIN cons.	0.738 ± 0.028	0.705	0.820
	D-MPNN cons.	0.762 ± 0.012	0.743	0.785
	GIN cons.	0.881 ± 0.045	0.825	0.969
logP	D-GIN	0.529 ± 0.064*	0.472	0.662
	D-MPNN	0.600 ± 0.063	0.540	0.734
	GIN	0.784 ± 0.077	0.716	0.901
	RF	0.681 ± 0.224	0.470	0.928
	SVM	0.693 ± 0.134	0.493	0.833
	KNN	1.014 ± 0.123	0.743	1.102
	D-GIN cons.	0.455 ± 0.028	0.428	0.515
	D-MPNN cons.	0.475 ± 0.027	0.443	0.532
	GIN cons.	0.566 ± 0.034	0.533	0.618

**Table 3:** Impact of the featurization and training strategy on the different molecular properties independent of what GNN model type was used. For each endpoint the mean, minimum, and maximum RMSE can be seen. The dark gray boxes show the best RMSE for the particular property, the light gray the second best.

Featurization		logD RMSE			logS RMSE			logP RMSE	
Strategy	Mean	Min	Max	Mean	Min	Max	Mean	Min	Max
3.0	0.689 ± 0.079	0.575	0.781	0.954 ± 0.146	0.831	1.177	0.596 ± 0.120	0.472	0.751
4.0	0.694 ± 0.072	0.587	0.791	0.948 ± 0.142	0.807	1.160	0.591 ± 0.105	0.487	0.725
5.0	0.813 ± 0.099	0.660	0.911	1.099 ± 0.180	0.938	1.361	0.760 ± 0.110	0.652	0.901
6.0	0.727 ± 0.132	0.553	0.900	1.015 ± 0.250	0.818	1.400	0.641 ± 0.183	0.493	0.883
7.0	0.732 ± 0.075	0.615	0.823	0.961 ± 0.113	0.851	1.144	0.629 ± 0.077	0.541	0.727
8.0	0.706 ± 0.083	0.579	0.814	0.970 ± 0.155	0.795	1.216	0.609 ± 0.114	0.477	0.750
**Training**		**logD RMSE**			**logS RMSE**			**logP RMSE**	
**Strategy**	**Mean**	**Min**	**Max**	**Mean**	**Min**	**Max**	**Mean**	**Min**	**Max**
logD/P/S	0.730 ± 0.102	0.579	0.900	0.979 ± 0.166	0.795	1.325	0.639 ± 0.129	0.487	0.901
logD/P	0.719 ± 0.105	0.553	0.911	-	-	-	-	-	-
logD/S	0.737 ± 0.106	0.582	0.911	0.993 ± 0.176	0.821	1.359	-	-	-
logS/P	-	-	-	0.988 ± 0.173	0.812	1.333	-	-	-
logD	0.722 ± 0.087	0.596	0.881	-	-	-	-	-	-
logS	-	-	-	1.004 ± 0.187	0.818	1.400	-	-	-
logP	-	-	-	-	-	-	0.637 ± 0.130	0.472	0.894

**Table 4:** Impact of the featurization on the different molecular properties. For each property and model type, the mean, minimum, and maximum RMSE are shown. The dark gray boxes represent the best RMSE for the particular property and model type, the light gray the second best. The red asterisk is the overall best RMSE for the particular property.

Model Type	Featurization		logD RMSE			logS RMSE			logP RMSE	
	Strategy	Mean	Min	Max	Mean	Min	Max	Mean	Min	Max
D-GIN	3.0	0.585 ± 0.011	0.575	0.601	0.835 ± 0.007	0.831	0.847	0.484 ± 0.017 *	0.472	0.496
	4.0	0.603 ± 0.014	0.587	0.622	0.827 ± 0.018	0.807	0.851	0.490 ± 0.004	0.487	0.493
	5.0	0.682 ± 0.018	0.660	0.704	1.007 ± 0.039	0.969	1.061	0.657 ± 0.006	0.652	0.662
	6.0	0.580 ± 0.019 *	0.553	0.596	0.825 ± 0.008 *	0.818	0.836	0.502 ± 0.012	0.493	0.511
	7.0	0.637 ± 0.020	0.615	0.661	0.880 ± 0.025	0.851	0.906	0.550 ± 0.013	0.541	0.560
	8.0	0.602 ± 0.020	0.579	0.629	0.826 ± 0.027	0.795	0.854	0.493 ± 0.023	0.477	0.509
D-MPNN	3.0	0.728 ± 0.023	0.703	0.759	0.835 ± 0.007	0.831	0.847	0.561 ± 0.012	0.552	0.570
	4.0	0.715 ± 0.016	0.692	0.728	0.879 ± 0.017	0.862	0.896	0.563 ± 0.010	0.556	0.570
	5.0	0.878 ± 0.023	0.857	0.911	0.951 ± 0.011	0.938	0.961	0.725 ± 0.012	0.716	0.734
	6.0	0.712 ± 0.017	0.686	0.724	0.868 ± 0.011	0.857	0.879	0.545 ± 0.007	0.540	0.551
	7.0	0.805 ± 0.016	0.783	0.823	0.892 ± 0.018	0.865	0.905	0.616 ± 0.017	0.604	0.629
	8.0	0.734 ± 0.036	0.712	0.788	0.911 ± 0.007	0.905	0.921	0.588 ± 0.007	0.581	0.593
GIN	3.0	0.755 ± 0.018	0.741	0.781	1.149 ± 0.022	1.122	1.177	0.745 ± 0.009	0.738	0.751
	4.0	0.765 ± 0.019	0.743	0.791	1.137 ± 0.020	1.116	1.160	0.720 ± 0.006	0.716	0.725
	5.0	0.880 ± 0.026	0.856	0.911	1.339 ± 0.017	1.325	1.361	0.897 ± 0.005	0.894	0.901
	6.0	0.889 ± 0.019	0.860	0.900	1.351 ± 0.037	1.314	1.400	0.877 ± 0.008	0.871	0.883
	7.0	0.755 ± 0.007	0.747	0.765	1.112 ± 0.028	1.088	1.144	0.722 ± 0.007	0.716	0.727
	8.0	0.781 ± 0.033	0.734	0.814	1.172 ± 0.033	1.135	1.216	0.745 ± 0.006	0.741	0.750

**Table 5:** Impact of the training strategies on the different molecular properties. Each model type is evaluated separately. For each property and model type, the mean, minimum, and maximum RMSE are shown. The dark gray boxes represent the best RMSE for the particular property and model type, the light gray the second best. The red asterisk highlights the overall best RMSE for the particular property.

Model Type	Training		logD RMSE			logS RMSE			logP RMSE	
	Strategy	Mean	Min	Max	Mean	Min	Max	Mean	Min	Max
D-GIN	logD/P/S	0.617 ± 0.052	0.579	0.704	0.851 ± 0.071 *	0.795	0.987	0.534 ± 0.067	0.487	0.662
	logD/P	0.607 ± 0.044 *	0.553	0.685	-	-	-	-	-	-
	logD/S	0.610 ± 0.036	0.582	0.679	0.875 ± 0.072	0.821	1.010	-	-	-
	logS/P	-	-	-	0.868 ± 0.095	0.812	1.061	-	-	-
	logD	0.625 ± 0.024	0.596	0.660	-	-	-	-	-	-
	logS	-	-	-	0.872 ± 0.053	0.818	0.969	-	-	-
	logP	-	-	-	-	-	-	0.524 ± 0.067 *	0.472	0.652
D-MPNN	logD/P/S	0.759 ± 0.063	0.712	0.864	0.903 ± 0.031	0.873	0.961	0.596 ± 0.062	0.551	0.716
	logD/P	0.741 ± 0.066	0.686	0.857	-	-	-	-	-	-
	logD/S	0.788 ± 0.070	0.721	0.911	0.892 ± 0.039	0.857	0.960	-	-	-
	logS/P	-	-	-	0.894 ± 0.029	0.860	0.938	-	-	-
	logD	0.761 ± 0.067	0.713	0.881	-	-	-	-	-	-
	logS	-	-	-	0.897 ± 0.030	0.865	0.944	-	-	-
	logP	-	-	-	-	-	-	0.603 ± 0.071	0.540	0.734
GIN	logD/P/S	0.815 ± 0.063	0.756	0.901	1.183 ± 0.106	1.089	1.325	0.786 ± 0.083	0.716	0.901
	logD/P	0.809 ± 0.075	0.742	0.911	-	-	-	-	-	-
	logD/S	0.811 ± 0.056	0.751	0.896	1.213 ± 0.108	1.126	1.359	-	-	-
	logS/P	-	-	-	1.201 ± 0.102	1.088	1.333	-	-	-
	logD	0.780 ± 0.060	0.734	0.860	-	-	-	-	-	-
	logS	-	-	-	1.243 ± 0.109	1.144	1.400	-	-	-
	logP	-	-	-	-	-	-	0.782 ± 0.077	0.725	0.894

**Figure A1:**
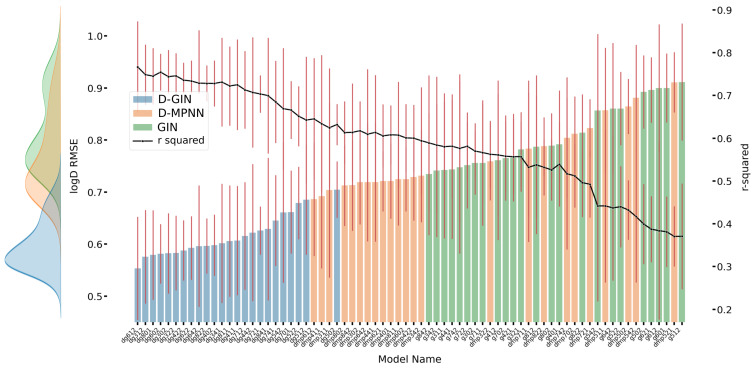
The first y-axis specifies the logD RMSE and the secondary x-axis the corresponding $r^{2}$ values for each GNN model. The D-GIN is colored blue, the D-MPNN orange and the GIN green. Each of the boxes represents one different model run. The kernel density of each model is shown on the very left side. The red lines correspond to the 95% confidence intervals. The model names are a combination of model type (D-GIN, GIN, D-MPNN), training approach and featurization type-a detailed description of each model name can be found in Table A8, Table A9, Table A10, Table A11, Table A12, Table A13, Table A14, Table A15, Table A16, Table A17, Table A18 and Table A19 in the Appendix A.

**Figure A2:**
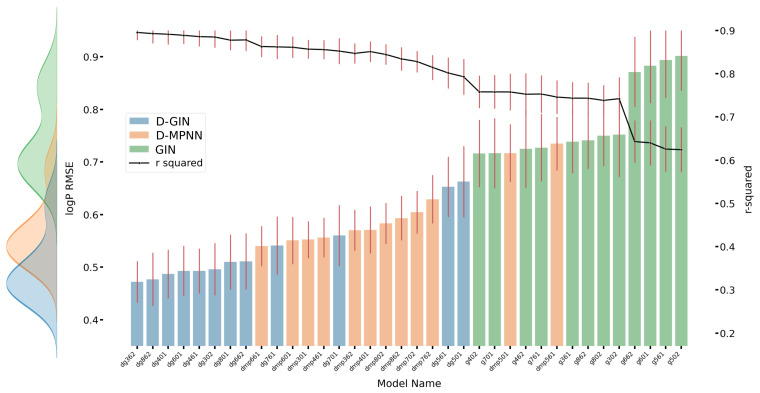
The first y-axis specifies the logP RMSE and the secondary x-axis the corresponding $r^{2}$ values for each GNN model. The D-GIN is colored blue, the D-MPNN orange and the GIN green. Each of the boxes represents one different model run. The kernel density of each model is shown on the very left side. The red lines correspond to the 95% confidence intervals. The model names are a combination of model type (D-GIN, GIN, D-MPNN), training approach and featurization type-a detailed description of each model name can be found in Table A8, Table A9, Table A10, Table A11, Table A12, Table A13, Table A14, Table A15, Table A16, Table A17, Table A18 and Table A19 in the Appendix A.

**Figure A3:**
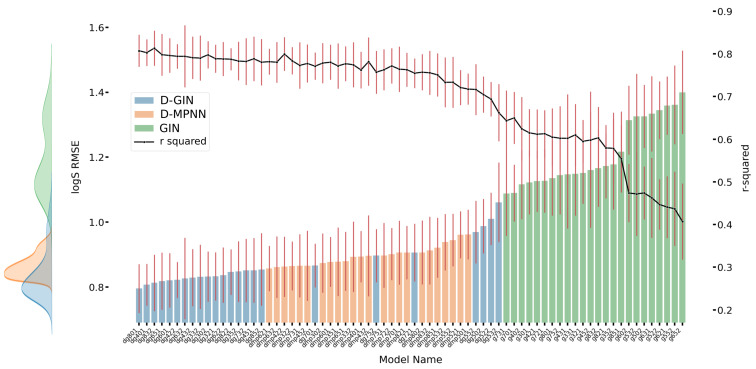
The first y-axis specifies the logS RMSE and the secondary x-axis the corresponding $r^{2}$ values for each GNN model. The D-GIN is colored blue, the D-MPNN orange and the GIN green. Each of the boxes represents one different model run. The kernel density of each model is shown on the very left side. The red lines correspond to the 95% confidence intervals. The model names are a combination of model type (D-GIN, GIN, D-MPNN), training approach and featurization type-a detailed description of each model name can be found in Table A8, Table A9, Table A10, Table A11, Table A12, Table A13, Table A14, Table A15, Table A16, Table A17, Table A18 and Table A19 in the Appendix A.

## Data Availability

Python package used in this work (release v0.1): https://github.com/spudlig/graph_networks. Data is available on https://zenodo.org/record/5137613#.YQortyWxVhG and was accessed on 26 July 2021.

## Appendix A

The appendix includes informational materials that show the featurization of the GNN and non-GNN baseline models in Table A7, in Figure A1 and Figure A2 the individual models and their corresponding names can be seen. These are the same Figures as in the main body but include the unique identifiers. These identifiers show what kind of model type, featurization and training approach was used when looked up in Table A8, Table A9, Table A10, Table A11, Table A12, Table A13, Table A14, Table A15, Table A16, Table A17, Table A18 and Table A19. The run-time to fit the used RF, SVM, and KNN models with 3380 compounds on logD is approximately 50 s (RF), 25 s (SVM), and 0.5 s (KNN). When training the GNN model types on the 3380 logD compounds it takes for each epoch approximately 56 s (D-GIN), 35 s (D-MPNN), and 28 s (GIN).

**Table A1:** Node and edge featurization of type 3. Each node or edge featurization vector consisted of a concatenation of the different one-hot encoded or floating point feature vectors according to their possible states (if present) and corresponding size-36 for nodes and 20 for edges.

Feature	Possible States	Size
chemical element	H, C, N, O, S, F, P, Cl, Br, I	10
calculated formal charge	−2, −1, 0, 1, 2	5
CIP configuration	R, S, None, either	4
hybridization state	sp, sp2, sp3, sp3d, sp3d2, none	6
amide center	yes, no	2
present in aromatic ring	yes, no	2
ring size	1/size	1
nr. of hydrogens	0, 1, 2, 3, 4, 5	6
bond order	1, 2, 3	3
conjugated	yes, no	2
rotate−able	yes, no	2
amide bond	yes, no	2
present in aromatic ring	yes, no	2
present in ring	yes, no	2
ring size	1/size	1
CIP configuration	none, E, Z, trans, cis, either	6

**Table A2:** Node and edge featurization of type 4. Each node or edge featurization vector consisted of a concatenation of the different one-hot encoded or floating point feature vectors according to their possible states (if present) and corresponding size-45 for nodes and 29 for edges.

Feature	Possible States	Size
chemical element	H, C, N, O, S, F, P, Cl, Br, I	10
calculated formal charge	−2, −1, 0, 1, 2	5
CIP configuration	R, S, None, either	4
hybridization state	sp, sp2, sp3, sp3d, sp3d2, none	6
amide center	yes, no	2
present in aromatic ring	yes, no	2
ring size	0, 3, 4, 5, 6, 7, 8, 9, 10, 11	1
nr. of hydrogens	0, 1, 2, 3, 4, 5	6
bond order	1, 2, 3	3
conjugated	yes, no	2
rotate-able	yes, no	2
amide bond	yes, no	2
present in aromatic ring	yes, no	2
present in ring	yes, no	2
ring size	0, 3, 4, 5, 6, 7, 8, 9, 10, 11	1
CIP configuration	none, E, Z, trans, cis, either	6

**Table A3:** Node and edge featurization of type 5. Each node or edge featurization vector consisted of a concatenation of the different one-hot encoded or floating point feature vectors according to their possible states (if present) and corresponding size-15 for nodes and 3 for edges.

Feature	Possible States	Size
chemical element	H, C, N, O, S, F, P, Cl, Br, I	10
calculated formal charge	−2, −1, 0, 1, 2	5
CIP configuration	-	-
hybridization state	-	-
amide center	-	-
present in aromatic ring	-	-
ring size	-	-
nr. of hydrogens	-	-
bond order	1, 2, 3	3
conjugated	-	-
rotate-able	-	-
amide bond	-	-
present in aromatic ring	-	-
present in ring	-	-
ring size	-	-
CIP configuration	-	-

**Table A4:** Node and edge featurization of type 6. Each node or edge featurization vector consisted of a concatenation of the different one-hot encoded or floating point feature vectors according to their possible states (if present) and corresponding size-15 for nodes and 20 for edges.

Feature	Possible States	Size
chemical element	H, C, N, O, S, F, P, Cl, Br, I	10
calculated formal charge	−2, −1, 0, 1, 2	5
CIP configuration	-	-
hybridization state	-	-
amide center	-	-
present in aromatic ring	-	-
present in ring	-	-
nr. of hydrogens	-	-
bond order	1, 2, 3	3
conjugated	yes, no	2
rotate-able	yes, no	2
amide bond	yes, no	2
present in aromatic ring	yes, no	2
present in ring	yes, no	2
ring size	1/size	1
CIP configuration	none, E, Z, trans, cis, either	6

**Table A5:** Node and edge featurization of type 7. Each node or edge featurization vector consisted of a concatenation of the different one-hot encoded or floating point feature vectors according to their possible states (if present) and corresponding size-36 for nodes and 3 for edges.

Feature	Possible States	Size
chemical element	H, C, N, O, S, F, P, Cl, Br, I	10
calculated formal charge	−2, −1, 0, 1, 2	5
CIP configuration	R, S, None, either	4
hybridization state	sp, sp2, sp3, sp3d, sp3d2, none	6
amide center	yes, no	2
present in aromatic ring	yes, no	2
ring size	0, 3, 4, 5, 6, 7, 8, 9, 10, 11	1
nr. of hydrogens	0, 1, 2, 3, 4, 5	6
bond order	1, 2, 3	3
conjugated	-	-
rotate-able	-	-
amide bond	-	-
present in aromatic ring	-	-
present in ring	-	-
ring size	-	-
CIP configuration	-	-

**Table A6:** Node and edge featurization of type 8. Each node or edge featurization vector consisted of a concatenation of the different one-hot encoded or floating point feature vectors according to their possible states (if present) and corresponding size-26 for nodes and 7 for edges.

Feature	Possible States	Size
chemical element	H, C, N, O, S, F, P, Cl, Br, I	10
formal charge	−2, −1, 0, 1, 2	5
CIP priority rule	R, S, None, either	4
hybridization state	sp, sp2, sp3, sp3d, sp3d2, none	6
amide center	-	-
aromaticity	-	-
ring size	float (1/size)	1
nr. of hydrogens	-	-
bond order	1, 2, 3	3
conjugated	-	-
rotate-able	yes, no	2
amide bond	-	-
aromaticity	-	-
present in ring	yes, no	2
ring size	-	-
CIP priority rule	-	-

**Table A7:** Non-GNN featurization. The identifier is used as reference.

Identifier	Fingerprint	Radius	nr. Bits	Descriptor
10	ECFP	4	1024	No
11	ECFP	4	1536	No
12	ECFP	4	2048	No
13	MACCSKeys	-	-	No
14	ECFP	4	1024	Yes
15	ECFP	4	1536	Yes
16	ECFP	4	2048	Yes
17	MACCSKeys	-	-	Yes

**Table A8:** Shows the log D RMSE and $r^{2}$ results for the D-GIN model type used during this survey. The last column consist of the unique identify. Training strategy 0 represents a training strategy combining logD, logS, and logP, 1 represents a strategy combining logD and logP, 2 stands for a combination of logD and logS, 3 represents a strategy using logS and logP, 4 represents a strategy using logD, 5 represents a strategy using logS, and 6 represents a strategy using logP.

Model	Training	Featurization	logD	$r^{2}$	Unique
Type	Strategy	Strategy	RMSE		ID
D-GIN	2	5	0.679 ± 0.034	0.652 ± 0.035	dg522
D-GIN	2	8	0.596 ± 0.026	0.728 ± 0.021	dg822
D-GIN	2	4	0.587 ± 0.029	0.736 ± 0.020	dg422
D-GIN	2	3	0.582 ± 0.035	0.746 ± 0.027	dg322
D-GIN	0	3	0.582 ± 0.038	0.744 ± 0.031	dg302
D-GIN	0	6	0.581 ± 0.028	0.755 ± 0.021	dg602
D-GIN	0	4	0.598 ± 0.029	0.728 ± 0.027	dg402
D-GIN	4	3	0.601 ± 0.057	0.731 ± 0.052	dg341
D-GIN	0	8	0.579 ± 0.043	0.745 ± 0.033	dg801
D-GIN	1	3	0.575 ± 0.044	0.749 ± 0.035	dg312
D-GIN	1	8	0.605 ± 0.053	0.722 ± 0.046	dg811
D-GIN	4	6	0.596 ± 0.058	0.729 ± 0.062	dg642
D-GIN	1	4	0.606 ± 0.052	0.725 ± 0.053	dg411
D-GIN	1	7	0.615 ± 0.051	0.713 ± 0.045	dg712
D-GIN	4	4	0.622 ± 0.065	0.707 ± 0.065	dg442
D-GIN	2	7	0.626 ± 0.023	0.704 ± 0.022	dg721
D-GIN	4	8	0.629 ± 0.068	0.700 ± 0.068	dg841
D-GIN	4	7	0.645 ± 0.043	0.685 ± 0.048	dg741
D-GIN	4	5	0.660 ± 0.067	0.669 ± 0.071	dg542
D-GIN	0	7	0.661 ± 0.039	0.666 ± 0.034	dg701
D-GIN	1	5	0.685 ± 0.052	0.643 ± 0.074	dg512
D-GIN	1	6	0.553 ± 0.049	0.767 ± 0.053	dg612
D-GIN	0	5	0.704 ± 0.027	0.632 ± 0.024	dg502
D-GIN	2	6	0.592 ± 0.030	0.734 ± 0.024	dg622
D-GIN cons.	2	5	0.605 ± 0.032	0.719 ± 0.031	dg522_cons
D-GIN cons.	0	5	0.622 ± 0.030	0.704 ± 0.027	dg502_cons
D-GIN cons.	0	7	0.603 ± 0.030	0.722 ± 0.025	dg701_cons
D-GIN cons.	1	5	0.609 ± 0.056	0.714 ± 0.062	dg512_cons
D-GIN cons.	1	6	0.548 ± 0.051	0.769 ± 0.049	dg612_cons
D-GIN cons.	4	7	0.589 ± 0.045	0.734 ± 0.043	dg741_cons
D-GIN cons.	1	3	0.561 ± 0.047	0.758 ± 0.039	dg312_cons
D-GIN cons.	2	4	0.557 ±0.029	0.762 ± 0.023	dg422_cons
D-GIN cons.	4	3	0.557 ± 0.052	0.762 ± 0.044	dg341_cons
D-GIN cons.	2	3	0.549 ± 0.034	0.769 ± 0.026	dg322_cons
D-GIN cons.	4	5	0.590 ± 0.059	0.733 ± 0.055	dg542_cons
D-GIN cons.	0	8	0.562 ± 0.032	0.758 ± 0.025	dg801_cons
D-GIN cons.	0	4	0.563 ± 0.031	0.757 ± 0.027	dg402_cons
D-GIN cons.	1	7	0.566 ± 0.055	0.754 ± 0.044	dg712_cons
D-GIN cons.	1	4	0.567 ± 0.051	0.754 ± 0.041	dg411_cons
D-GIN cons.	0	3	0.562 ± 0.033	0.758 ± 0.024	dg302_cons
D-GIN cons.	4	8	0.575 ± 0.052	0.746 ± 0.053	dg841_cons
D-GIN cons.	2	8	0.568 ± 0.030	0.753 ± 0.026	dg821_cons
D-GIN cons.	2	7	0.580 ± 0.027	0.742 ± 0.023	dg721_cons
D-GIN cons.	1	8	0.580 ± 0.053	0.742 ± 0.047	dg811_cons
D-GIN cons.	4	4	0.577 ± 0.052	0.744 ± 0.041	dg442_cons
D-GIN cons.	4	6	0.568 ± 0.054	0.751 ± 0.051	dg642_cons
D-GIN cons.	0	6	0.569 ± 0.029	0.751 ± 0.024	dg602_cons
D-GIN cons.	2	6	0.571 ± 0.031	0.750 ± 0.024	dg622_cons

**Table A9:** Shows the logD RMSE and $r^{2}$ results for the D-MPNN model type used during this survey. The last column consists of the unique identifier. Training strategy 0 represents a training strategy combining logD, logS, and logP, 1 represents a strategy combining logD and logP, 2 stands for a combination of logD and logS, 3 represents a strategy using logS and logP, 4 represents a strategy using logD, 5 represents a strategy using logS, and 6 represents a strategy using logP.

Model	Training	Featurization	logD	$r^{2}$	Unique
Type	Strategy	Strategy	RMSE		ID
D-MPNN	1	7	0.7836 ± 0.065	0.532 ± 0.087	dmp711
D-MPNN	1	6	0.686 ± 0.054	0.645 ± 0.071	dmp612
D-MPNN	1	5	0.857 ± 0.060	0.442 ± 0.089	dmp511
D-MPNN	2	3	0.759 ± 0.037	0.563 ± 0.039	dmp322
D-MPNN	1	4	0.692 ± 0.069	0.634 ± 0.080	dmp411
D-MPNN	2	5	0.911 ± 0.029	0.371 ± 0.035	dmp521
D-MPNN	2	6	0.721 ± 0.029	0.606 ± 0.037	dmp621
D-MPNN	0	4	0.721 ± 0.036	0.608 ± 0.034	dmp401
D-MPNN	0	8	0.712 ± 0.039	0.613 ± 0.037	dmp802
D-MPNN	4	8	0.713 ± 0.043	0.614 ± 0.057	dmp842
D-MPNN	1	8	0.724 ± 0.044	0.607 ± 0.062	dmp811
D-MPNN	4	4	0.719 ± 0.057	0.614 ± 0.067	dmp441
D-MPNN	4	6	0.719 ± 0.056	0.610 ± 0.076	dmp642
D-MPNN	4	3	0.731 ± 0.044	0.594 ± 0.062	dmp342
D-MPNN	0	6	0.724 ± 0.030	0.601 ± 0.040	dmp602
D-MPNN	1	3	0.703 ± 0.084	0.625 ± 0.069	dmp311
D-MPNN	0	3	0.719 ± 0.040	0.618 ± 0.034	dmp302
D-MPNN	2	8	0.788 ± 0.027	0.532 ± 0.033	dmp822
D-MPNN	2	4	0.728 ± 0.041	0.600 ± 0.039	dmp422
D-MPNN	2	7	0.823 ± 0.027	0.493 ± 0.039	dmp721
D-MPNN	4	7	0.804 ± 0.058	0.517 ± 0.089	dmp742
D-MPNN	4	5	0.881 ± 0.051	0.417 ± 0.077	dmp542
D-MPNN	0	7	0.812 ± 0.035	0.512 ± 0.037	dmp702
D-MPNN	0	5	0.864 ± 0.026	0.432 ± 0.035	dmp502
D-MPNN cons.	0	4	0.633 ± 0.031	0.693 ± 0.025	dmp402_cons
D-MPNN cons.	4	5	0.699 ± 0.047	0.625 ± 0.056	dmp542_cons
D-MPNN cons.	2	4	0.632 ± 0.031	0.694 ± 0.028	dmp422_cons
D-MPNN cons.	2	6	0.632 ± 0.029	0.694 ± 0.028	dmp621_cons
D-MPNN cons.	2	5	0.710 ± 0.027	0.614 ± 0.024	dmp521_cons
D-MPNN cons.	1	8	0.632 ± 0.050	0.693 ± 0.058	dmp811_cons
D-MPNN cons.	0	3	0.625 ± 0.030	0.701 ± 0.026	dmp302_cons
D-MPNN cons.	4	4	0.625 ± 0.054	0.700 ± 0.052	dmp441_cons
D-MPNN cons.	1	3	0.625 ± 0.057	0.700 ± 0.050	dmp311_cons
D-MPNN cons.	0	8	0.624 ± 0.029	0.701 ± 0.023	dmp802_cons
D-MPNN cons.	4	8	0.622 ± 0.045	0.703 ± 0.050	dmp842_cons
D-MPNN cons.	1	6	0.618 ± 0.045	0.706 ± 0.051	dmp612_cons
D-MPNN cons.	1	4	0.613 ± 0.055	0.711 ± 0.057	dmp411_cons
D-MPNN cons.	4	6	0.634 ± 0.055	0.691 ± 0.066	dmp642_cons
D-MPNN cons.	0	6	0.636 ± 0.030	0.690 ± 0.029	dmp602_cons
D-MPNN cons.	2	3	0.646 ± 0.032	0.680 ± 0.028	dmp322_cons
D-MPNN cons.	1	5	0.688 ± 0.059	0.637 ± 0.057	dmp512_cons
D-MPNN cons.	2	8	0.652 ± 0.030	0.674 ± 0.025	dmp822_cons
D-MPNN cons.	1	7	0.654 ± 0.060	0.671 ± 0.069	dmp711_cons
D-MPNN cons.	4	7	0.663 ± 0.045	0.662 ± 0.058	dmp742_cons
D-MPNN cons.	0	7	0.670 ± 0.028	0.656 ± 0.026	dmp701_cons
D-MPNN cons.	2	7	0.674 ± 0.026	0.652 ± 0.029	dmp721_cons
D-MPNN cons.	0	5	0.697 ± 0.026	0.628 ± 0.024	dmp502_cons
D-MPNN cons.	4	3	0.636 ± 0.042	0.689 ± 0.055	dmp342_cons

**Table A10:** Shows the logD RMSE and $r^{2}$ results for the GIN model type used during this survey. The last column consist of the unique identify. Training strategy 0 represents a training strategy combining logD, logS, and logP, 1 represents a strategy combining logD and logP, 2 stands for a combination of logD and logS, 3 represents a strategy using logS and logP, 4 represents a strategy using logD, 5 represents a strategy using logS, and 6 represents a strategy using logP.

Model	Training	Featurization	logD	$r^{2}$	Unique
Type	Strategy	Strategy	RMSE		ID
GIN	2	5	0.860 ± 0.035	0.440 ± 0.047	g522
GIN	2	6	0.896 ± 0.031	0.387 ± 0.039	g621
GIN	0	4	0.791 ± 0.051	0.539 ± 0.050	g401
GIN	0	8	0.789 ± 0.031	0.526 ± 0.036	g802
GIN	2	8	0.814 ± 0.036	0.496 ± 0.040	g822
GIN	1	8	0.786 ± 0.068	0.538 ± 0.081	g812
GIN	1	6	0.899 ± 0.061	0.384 ± 0.104	g612
GIN	4	8	0.734 ± 0.058	0.589 ± 0.079	g842
GIN	4	5	0.856 ± 0.073	0.442 ± 0.112	g542
GIN	0	5	0.892 ± 0.035	0.400 ± 0.047	g502
GIN	4	3	0.741 ± 0.063	0.584 ± 0.080	g342
GIN	1	7	0.756 ± 0.060	0.567 ± 0.057	g711
GIN	1	4	0.761 ± 0.076	0.561 ± 0.071	g412
GIN	4	4	0.743 ± 0.066	0.581 ± 0.061	g441
GIN	4	7	0.747 ± 0.082	0.576 ± 0.087	g742
GIN	2	7	0.751 ± 0.041	0.581 ± 0.040	g722
GIN	2	3	0.781 ± 0.035	0.557 ± 0.041	g321
GIN	2	4	0.766 ± 0.042	0.557 ± 0.044	g421
GIN	0	7	0.765 ± 0.040	0.558 ± 0.041	g702
GIN	0	3	0.756 ± 0.030	0.570 ± 0.033	g302
GIN	1	3	0.742 ± 0.065	0.580 ± 0.062	g311
GIN	4	6	0.860 ± 0.063	0.437 ± 0.085	g642
GIN	0	6	0.900 ± 0.033	0.381 ± 0.041	g601
GIN	1	5	0.911 ± 0.056	0.371 ± 0.062	g512
GINcons.	0	6	0.715 ± 0.020	0.609 ± 0.025	g602_cons
GINcons.	4	3	0.627 ± 0.054	0.698 ± 0.059	g342_cons
GINcons.	4	8	0.632 ± 0.050	0.693 ± 0.057	g842_cons
GINcons.	4	7	0.633 ± 0.061	0.692 ± 0.056	g742_cons
GINcons.	4	4	0.637 ± 0.058	0.688 ± 0.060	g441_cons
GINcons.	1	3	0.640 ± 0.056	0.685 ± 0.051	g311_cons
GINcons.	2	7	0.642 ± 0.033	0.685 ± 0.028	g722_cons
GINcons.	1	7	0.644 ± 0.047	0.682 ± 0.053	g711_cons
GINcons.	2	4	0.645 ± 0.034	0.681 ± 0.031	g421_cons
GINcons.	0	3	0.647 ± 0.029	0.679 ± 0.025	g302_cons
GINcons.	1	4	0.648 ± 0.059	0.678 ± 0.056	g412_cons
GINcons.	0	4	0.652 ± 0.033	0.674 ± 0.030	g401_cons
GINcons.	0	7	0.654 ± 0.029	0.673 ± 0.024	g701_cons
GINcons.	2	3	0.656 ± 0.028	0.670 ± 0.030	g321_cons
GINcons.	1	8	0.657 ± 0.055	0.669 ± 0.068	g812_cons
GINcons.	0	8	0.661 ± 0.034	0.665 ± 0.031	g801_cons
GINcons.	2	8	0.673 ± 0.032	0.653 ± 0.029	g822_cons
GINcons.	4	5	0.689 ± 0.056	0.636 ± 0.064	g542_cons
GINcons.	4	6	0.692 ± 0.052	0.633 ± 0.057	g642_cons
GINcons.	1	6	0.707 ± 0.057	0.616 ± 0.061	g612_cons
GINcons.	2	6	0.707 ± 0.025	0.617 ± 0.025	g622_cons
GINcons.	0	5	0.712 ± 0.033	0.612 ± 0.030	g502_cons
GINcons.	1	5	0.719 ± 0.046	0.603 ± 0.052	g512_cons
GINcons.	2	5	0.692 ± 0.026	0.634 ± 0.022	g522_cons

**Table A11:** Shows the logD RMSE and $r^{2}$ results for the non-GNN model types used during this survey. The last column consists of the unique identifier. Training strategy 0 represents a training strategy combining logD, logS, and logP, 1 represents a strategy combining logD and logP, 2 stands for a combination of logD and logS, 3 represents a strategy using logS and logP, 4 represents a strategy using logD, 5 represents a strategy using logS, and 6 represents a strategy using logP.

Model	Training	Featurization	logD	$r^{2}$	Unique
Type	Strategy	Strategy	RMSE		ID
KNN	logD	10	0.979 ± 0.072	0.261 ± 0.094	KNN10
KNN	logD	16	0.970 ± 0.056	0.275 ± 0.097	KNN16
KNN	logD	12	0.959 ± 0.066	0.293 ± 0.080	KNN12
KNN	logD	11	0.993 ± 0.072	0.240 ± 0.096	KNN11
KNN	logD	13	0.909 ± 0.067	0.363 ± 0.069	KNN13
KNN	logD	15	1.003 ± 0.057	0.226 ± 0.096	KNN15
KNN	logD	14	0.996 ± 0.065	0.236 ± 0.110	KNN14
KNN	logD	17	0.801 ± 0.060	0.506 ± 0.058	KNN17
RF	logD	17	0.708 ± 0.060	0.614 ± 0.055	rf17
RF	logD	15	0.699 ± 0.062	0.623 ± 0.059	rf15
RF	logD	16	0.703 ± 0.061	0.620 ± 0.057	rf16
RF	logD	11	0.859 ± 0.062	0.433 ± 0.062	rf11
RF	logD	14	0.706 ± 0.062	0.616 ± 0.058	rf14
RF	logD	10	0.890 ± 0.060	0.390 ± 0.062	rf10
RF	logD	13	0.813 ± 0.060	0.491 ± 0.059	rf13
RF	logD	12	0.863 ± 0.068	0.427 ± 0.068	rf12
SVM	logD	12	0.782 ± 0.061	0.529 ± 0.055	svm12
SVM	logD	16	0.707 ± 0.056	0.615 ± 0.047	svm16
SVM	logD	10	0.810 ± 0.062	0.495 ± 0.057	svm10
SVM	logD	15	0.698 ± 0.055	0.625 ± 0.045	svm15
SVM	logD	14	0.674 ± 0.054	0.650 ± 0.043	svm14
SVM	logD	17	0.639 ± 0.051	0.686 ± 0.046	svm17
SVM	logD	11	0.793 ± 0.060	0.516 ± 0.060	svm11
SVM	logD	13	0.814 ± 0.059	0.490 ± 0.055	svm13

**Table A12:** Shows the logS RMSE and $r^{2}$ results for the D-GIN model type used during this survey. The last column consists of the unique identifier. Training strategy 0 represents a training strategy combining logD, logS, and logP, 1 represents a strategy combining logD and logP, 2 stands for a combination of logD and logS, 3 represents a strategy using logS and logP, 4 represents a strategy using logD, 5 represents a strategy using logS, and 6 represents a strategy using logP.

Model	Training	Featurization	logD	$r^{2}$	Unique
Type	Strategy	Strategy	RMSE		ID
D-GIN	5	7	0.897 ± 0.041	0.757 ± 0.025	dg752
D-GIN	0	7	0.866 ± 0.034	0.771 ± 0.016	dg701
D-GIN	5	5	0.969 ± 0.048	0.717 ± 0.030	dg552
D-GIN	0	5	0.988 ± 0.042	0.705 ± 0.021	dg502
D-GIN	2	5	1.010 ± 0.040	0.694 ± 0.020	dg522
D-GIN	0	8	0.795 ± 0.038	0.807 ± 0.019	dg801
D-GIN	0	4	0.807 ± 0.032	0.803 ± 0.016	dg401
D-GIN	3	8	0.813 ± 0.044	0.813 ± 0.021	dg832
D-GIN	5	6	0.818 ± 0.044	0.798 ± 0.025	dg651
D-GIN	5	3	0.848 ± 0.047	0.783 ± 0.023	dg352
D-GIN	0	6	0.821 ± 0.042	0.796 ± 0.020	dg601
D-GIN	2	7	0.906 ± 0.045	0.755 ± 0.017	dg721
D-GIN	2	4	0.822 ± 0.028	0.794 ± 0.014	dg422
D-GIN	3	6	0.827 ± 0.063	0.794 ± 0.036	dg632
D-GIN	5	8	0.854 ± 0.056	0.781 ± 0.027	dg852
D-GIN	3	4	0.829 ± 0.044	0.791 ± 0.025	dg432
D-GIN	3	3	0.831 ± 0.050	0.790 ± 0.026	dg332
D-GIN	0	3	0.832 ± 0.039	0.798 ± 0.016	dg302
D-GIN	2	3	0.833 ± 0.037	0.789 ± 0.016	dg321
D-GIN	5	4	0.852 ± 0.050	0.789 ± 0.026	dg451
D-GIN	3	7	0.851 ± 0.049	0.782 ± 0.027	dg732
D-GIN	2	6	0.837 ± 0.042	0.788 ± 0.020	dg622
D-GIN	3	5	1.061 ± 0.061	0.662 ± 0.034	dg532
D-GIN cons.	2	5	0.794 ± 0.039	0.807 ± 0.014	dg522_cons
D-GIN cons.	0	5	0.779 ± 0.040	0.814 ± 0.014	dg502_cons
D-GIN cons.	5	5	0.778 ± 0.049	0.816 ± 0.020	dg552_cons
D-GIN cons.	3	5	0.820 ± 0.054	0.795 ± 0.024	dg532_cons
D-GIN cons.	0	8	0.705 ± 0.039	0.848 ± 0.014	dg801_cons
D-GIN cons.	2	7	0.757 ± 0.044	0.825 ± 0.015	dg721_cons
D-GIN cons.	2	8	0.734 ± 0.033	0.835 ± 0.012	dg822_cons
D-GIN cons.	3	7	0.733 ± 0.046	0.836 ± 0.021	dg732_cons
D-GIN cons.	0	3	0.733 ± 0.040	0.836 ± 0.015	dg301_cons
D-GIN cons.	0	7	0.731 ± 0.035	0.836 ± 0.010	dg701_cons
D-GIN cons.	5	3	0.730 ± 0.047	0.838 ± 0.019	dg352_cons
D-GIN cons.	3	3	0.727 ± 0.048	0.839 ± 0.021	dg332_cons
D-GIN cons.	5	4	0.739 ± 0.052	0.834 ± 0.023	dg451_cons
D-GIN cons.	5	7	0.748 ± 0.045	0.830 ± 0.018	dg752_cons
D-GIN cons.	2	3	0.725 ± 0.041	0.839 ± 0.014	dg321_cons
D-GIN cons.	3	8	0.724 ± 0.046	0.840 ± 0.018	dg832_cons
D-GIN cons.	3	4	0.722 ± 0.047	0.841 ± 0.020	dg432_cons
D-GIN cons.	2	4	0.718 ± 0.031	0.842 ± 0.011	dg422_cons
D-GIN cons.	3	6	0.718 ± 0.047	0.843 ± 0.021	dg631_cons
D-GIN cons.	2	6	0.716 ± 0.038	0.843 ± 0.014	dg622_cons
D-GIN cons.	0	4	0.715 ± 0.035	0.843 ± 0.011	dg401_cons
D-GIN cons.	5	6	0.711 ± 0.046	0.846 ± 0.021	dg651_cons
D-GIN cons.	0	6	0.724 ± 0.038	0.839 ± 0.016	dg601_cons
D-GIN cons.	5	8	0.735 ± 0.053	0.835 ± 0.021	dg852_cons

**Table A13:** Shows the logS RMSE and $r^{2}$ results for the D-MPNN model type used during this survey. The last column consist of the unique identify. Training strategy 0 represents a training strategy combining logD, logS, and logP, 1 represents a strategy combining logD and logP, 2 stands for a combination of logD and logS, 3 represents a strategy using logS and logP, 4 represents a strategy using logD, 5 represents a strategy using logS, and 6 represents a strategy using logP.

Model	Training	Featurization	logD	$r^{2}$	Unique
Type	Strategy	Strategy	RMSE		ID
D-MPNN	3	8	0.913 ± 0.052	0.756 ± 0.026	dmp832
D-MPNN	2	6	0.857 ± 0.035	0.782 ± 0.015	dmp621
D-MPNN	2	3	0.865 ± 0.038	0.784 ± 0.016	dmp322
D-MPNN	0	5	0.962 ± 0.038	0.718 ± 0.018	dmp501
D-MPNN	0	8	0.907 ± 0.049	0.757 ± 0.021	dmp802
D-MPNN	5	8	0.921 ± 0.046	0.751 ± 0.025	dmp851
D-MPNN	2	8	0.906 ± 0.041	0.763 ± 0.020	dmp821
D-MPNN	0	7	0.906 ± 0.049	0.764 ± 0.027	dmp701
D-MPNN	2	7	0.902 ± 0.040	0.772 ± 0.021	dmp722
D-MPNN	3	5	0.938 ± 0.044	0.733 ± 0.029	dmp532
D-MPNN	5	5	0.945 ± 0.041	0.734 ± 0.030	dmp552
D-MPNN	3	4	0.896 ± 0.062	0.782 ± 0.028	dmp432
D-MPNN	0	4	0.893 ± 0.040	0.762 ± 0.022	dmp401
D-MPNN	3	3	0.893 ± 0.054	0.775 ± 0.026	dmp332
D-MPNN	5	6	0.879 ± 0.046	0.777 ± 0.021	dmp651
D-MPNN	5	3	0.878 ± 0.053	0.772 ± 0.027	dmp351
D-MPNN	0	6	0.877 ± 0.039	0.780 ± 0.021	dmp601
D-MPNN	0	3	0.874 ± 0.046	0.779 ± 0.024	dmp302
D-MPNN	2	5	0.961 ± 0.036	0.721 ± 0.021	dmp521
D-MPNN	5	4	0.866 ± 0.054	0.778 ± 0.022	dmp452
D-MPNN	3	7	0.865 ± 0.049	0.773 ± 0.027	dmp731
D-MPNN	2	4	0.863 ± 0.046	0.800 ± 0.020	dmp422
D-MPNN	5	7	0.897 ± 0.050	0.763 ± 0.024	dmp751
D-MPNN	3	6	0.861 ± 0.047	0.780 ± 0.024	dmp632
D-MPNN cons.	5	4	0.748 ± 0.055	0.830 ± 0.020	dmp452_cons
D-MPNN cons.	0	4	0.768 ± 0.040	0.819 ± 0.015	dmp401_cons
D-MPNN cons.	2	8	0.769 ± 0.039	0.819 ± 0.014	dmp821_cons
D-MPNN cons.	3	8	0.762 ± 0.052	0.823 ± 0.022	dmp832_cons
D-MPNN cons.	5	6	0.749 ± 0.053	0.829 ± 0.021	dmp652_cons
D-MPNN cons.	0	8	0.770 ± 0.044	0.818 ± 0.016	dmp802_cons
D-MPNN cons.	3	3	0.762 ± 0.052	0.823 ± 0.021	dmp332_cons
D-MPNN cons.	0	7	0.771 ± 0.044	0.818 ± 0.016	dmp701_cons
D-MPNN cons.	2	7	0.771 ± 0.040	0.818 ± 0.016	dmp722_cons
D-MPNN cons.	5	7	0.761 ± 0.051	0.824 ± 0.021	dmp751_cons
D-MPNN cons.	5	8	0.774 ± 0.048	0.817 ± 0.022	dmp851_cons
D-MPNN cons.	3	5	0.777 ± 0.048	0.816 ± 0.021	dmp532_cons
D-MPNN cons.	5	5	0.779 ± 0.045	0.815 ± 0.021	dmp552_cons
D-MPNN cons.	3	4	0.765 ± 0.056	0.822 ± 0.021	dmp431_cons
D-MPNN cons.	2	5	0.784 ± 0.038	0.811 ± 0.016	dmp521_cons
D-MPNN cons.	0	3	0.760 ± 0.042	0.823 ± 0.016	dmp302_cons
D-MPNN cons.	0	6	0.759 ± 0.039	0.823 ± 0.014	dmp601_cons
D-MPNN cons.	2	3	0.756 ± 0.039	0.825 ± 0.014	dmp322_cons
D-MPNN cons.	2	6	0.744 ± 0.038	0.831 ± 0.013	dmp621_cons
D-MPNN cons.	5	3	0.752 ± 0.055	0.828 ± 0.021	dmp351_cons
D-MPNN cons.	3	7	0.744 ± 0.053	0.831 ± 0.021	dmp731_cons
D-MPNN cons.	2	4	0.750 ± 0.044	0.828 ± 0.016	dmp422_cons
D-MPNN cons.	3	6	0.748 ± 0.055	0.830 ± 0.021	dmp632_cons
D-MPNN cons.	0	5	0.785 ± 0.040	0.811 ± 0.015	dmp501_cons

**Table A14:** Shows the logS RMSE and $r^{2}$ results for the GIN model type used during this survey. The last column consists of the unique identifier. Training strategy 0 represents a training strategy combining logD, logS, and logP, 1 represents a strategy combining logD and logP, 2 stands for a combination of logD and logS, 3 represents a strategy using logS and logP, 4 represents a strategy using logD, 5 represents a strategy using logS, and 6 represents a strategy using logP.

Model	Training	Featurization	logD	$r^{2}$	Unique
Type	Strategy	Strategy	RMSE		ID
GIN	2	4	1.126 ± 0.047	0.612 ± 0.029	g421
GIN	0	3	1.122 ± 0.049	0.615 ± 0.028	g301
GIN	0	4	1.116 ± 0.053	0.624 ± 0.037	g402
GIN	0	7	1.089 ± 0.044	0.650 ± 0.031	g701
GIN	3	7	1.088 ± 0.066	0.643 ± 0.036	g731
GIN	2	7	1.127 ± 0.049	0.613 ± 0.028	g721
GIN	3	4	1.147 ± 0.084	0.602 ± 0.052	g431
GIN	5	7	1.145 ± 0.060	0.602 ± 0.038	g752
GIN	5	6	1.400 ± 0.064	0.407 ± 0.045	g652
GIN	5	5	1.362 ± 0.061	0.437 ± 0.043	g552
GIN	2	6	1.359 ± 0.045	0.441 ± 0.036	g621
GIN	2	5	1.345 ± 0.044	0.447 ± 0.030	g522
GIN	3	6	1.334 ± 0.058	0.463 ± 0.047	g631
GIN	0	5	1.326 ± 0.049	0.474 ± 0.031	g502
GIN	0	8	1.136 ± 0.058	0.605 ± 0.034	g801
GIN	3	5	1.326 ± 0.066	0.472 ± 0.049	g532
GIN	5	8	1.217 ± 0.062	0.554 ± 0.040	g851
GIN	5	3	1.178 ± 0.070	0.579 ± 0.042	g352
GIN	2	8	1.173 ± 0.050	0.580 ± 0.027	g821
GIN	3	8	1.166 ± 0.059	0.603 ± 0.036	g832
GIN	5	4	1.161 ± 0.089	0.598 ± 0.057	g452
GIN	2	3	1.151 ± 0.047	0.595 ± 0.025	g321
GIN	3	3	1.148 ± 0.064	0.610 ± 0.036	g331
GIN	0	6	1.314 ± 0.053	0.474 ± 0.034	g602
GINcons.	5	6	0.969 ± 0.057	0.714 ± 0.029	g652_cons
GINcons.	2	7	0.840 ± 0.036	0.784 ± 0.014	g721_cons
GINcons.	5	5	0.945 ± 0.045	0.728 ± 0.023	g552_cons
GINcons.	0	3	0.842 ± 0.041	0.783 ± 0.016	g301_cons
GINcons.	5	4	0.861 ± 0.065	0.774 ± 0.031	g452_cons
GINcons.	2	8	0.867 ± 0.043	0.770 ± 0.017	g821_cons
GINcons.	5	3	0.872 ± 0.056	0.769 ± 0.026	g352_cons
GINcons.	3	3	0.857 ± 0.054	0.776 ± 0.024	g331_cons
GINcons.	0	7	0.825 ± 0.038	0.791 ± 0.016	g701_cons
GINcons.	5	8	0.887 ± 0.056	0.760 ± 0.026	g851_cons
GINcons.	2	3	0.855 ± 0.043	0.776 ± 0.015	g321_cons
GINcons.	5	7	0.854 ± 0.055	0.778 ± 0.026	g752_cons
GINcons.	3	7	0.828 ± 0.052	0.791 ± 0.023	g731_cons
GINcons.	0	4	0.835 ± 0.040	0.786 ± 0.016	g402_cons
GINcons.	3	4	0.860 ± 0.061	0.775 ± 0.029	g431_cons
GINcons.	2	4	0.835 ± 0.041	0.786 ± 0.014	g421_cons
GINcons.	0	6	0.926 ± 0.045	0.737 ± 0.017	g602_cons
GINcons.	0	5	0.933 ± 0.038	0.733 ± 0.015	g502_cons
GINcons.	3	5	0.934 ± 0.053	0.734 ± 0.025	g532_cons
GINcons.	2	5	0.938 ± 0.040	0.730 ± 0.015	g522_cons
GINcons.	3	6	0.940 ± 0.048	0.731 ± 0.025	g631_cons
GINcons.	2	6	0.945 ± 0.041	0.726 ± 0.017	g621_cons
GINcons.	0	8	0.850 ± 0.042	0.779 ± 0.014	g802_cons
GINcons.	3	8	0.861 ± 0.053	0.774 ± 0.018	g832_cons

**Table A15:** Shows the logS RMSE and $r^{2}$ results for the non-GNN model types used during this survey. The last column consists of the unique identifier. Training strategy 0 represents a training strategy combining logD, logS, and logP, 1 represents a strategy combining logD and logP, 2 stands for a combination of logD and logS, 3 represents a strategy using logS and logP, 4 represents a strategy using logD, 5 represents a strategy using logS, and 6 represents a strategy using logP.

Model	Training	Featurization	logD	$r^{2}$	Unique
Type	Strategy	Strategy	RMSE		ID
KNN	logS	14	1.547 ± 0.063	0.268 ± 0.054	KNN14
KNN	logS	11	1.587 ± 0.063	0.230 ± 0.054	KNN11
KNN	logS	10	1.587 ± 0.064	0.229 ± 0.056	KNN10
KNN	logS	12	1.600 ± 0.066	0.217 ± 0.055	KNN12
KNN	logS	13	1.280 ± 0.066	0.499 ± 0.036	KNN13
KNN	logS	15	1.676 ± 0.066	0.140 ± 0.068	KNN15
KNN	logS	17	1.058 ± 0.055	0.658 ± 0.032	KNN17
KNN	logS	16	1.670 ± 0.065	0.147 ± 0.061	KNN16
RF	logS	12	1.239 ± 0.056	0.530 ± 0.032	rf12
RF	logS	14	0.760 ± 0.043	0.823 ± 0.020	rf14
RF	logS	15	0.764 ± 0.044	0.821 ± 0.022	rf15
RF	logS	13	1.128 ± 0.061	0.611 ± 0.039	rf13
RF	logS	16	0.765 ± 0.045	0.821 ± 0.024	rf16
RF	logS	17	0.770 ± 0.049	0.818 ± 0.025	rf17
RF	logS	10	1.284 ± 0.055	0.495 ± 0.033	rf10
RF	logS	11	1.271 ± 0.057	0.506 ± 0.035	rf11
SVM	logS	12	1.142 ± 0.055	0.601 ± 0.035	svm12
SVM	logS	11	1.149 ± 0.057	0.596 ± 0.035	svm11
SVM	logS	16	0.966 ± 0.049	0.715 ± 0.024	svm16
SVM	logS	15	0.930 ± 0.046	0.735 ± 0.023	svm15
SVM	logS	13	1.086 ± 0.060	0.639 ± 0.034	svm13
SVM	logS	17	0.730 ± 0.041	0.837 ± 0.020	svm17
SVM	logS	14	0.891 ± 0.046	0.757 ± 0.023	svm14
SVM	logS	10	1.162 ± 0.056	0.587 ± 0.036	svm10

**Table A16:** Shows the logP RMSE and $r^{2}$ results for the D-GIN model type used during this survey. The last column consists of the unique identifier. Training strategy 0 represents a training strategy combining logD, logS, and logP, 1 represents a strategy combining logD and logP, 2 stands for a combination of logD and logS, 3 represents a strategy using logS and logP, 4 represents a strategy using logD, 5 represents a strategy using logS, and 6 represents a strategy using logP.

Model	Training	Featurization	logD	$r^{2}$	Unique
Type	Strategy	Strategy	RMSE		ID
D-GIN	0	8	0.510 ± 0.026	0.878 ± 0.012	dg801
D-GIN	0	3	0.496 ± 0.025	0.885 ± 0.012	dg302
D-GIN	0	6	0.493 ± 0.024	0.889 ± 0.010	dg601
D-GIN	6	4	0.493 ± 0.021	0.886 ± 0.011	dg461
D-GIN	6	7	0.541 ± 0.028	0.862 ± 0.014	dg761
D-GIN	0	4	0.487 ± 0.023	0.891 ± 0.012	dg401
D-GIN	6	8	0.477 ± 0.025	0.893 ± 0.011	dg862
D-GIN	0	7	0.560 ± 0.029	0.852 ± 0.015	dg701
D-GIN	0	5	0.663 ± 0.034	0.793 ± 0.021	dg501
D-GIN	6	5	0.653 ± 0.029	0.802 ± 0.018	dg561
D-GIN	6	3	0.472 ± 0.020	0.896 ± 0.009	dg362
D-GIN	6	6	0.511 ± 0.027	0.878 ± 0.013	dg662
D-GIN cons.	6	3	0.428 ± 0.026	0.914 ± 0.010	dg362 cons.
D-GIN cons.	6	5	0.502 ± 0.027	0.881 ± 0.013	dg561 cons.
D-GIN cons.	0	7	0.473 ± 0.032	0.894 ± 0.013	dg701 cons.
D-GIN cons.	0	5	0.515 ± 0.033	0.875 ± 0.014	dg501 cons.
D-GIN cons.	0	3	0.447 ± 0.030	0.906 ± 0.012	dg302 cons.
D-GIN cons.	6	8	0.433 ± 0.030	0.912 ± 0.012	dg862 cons.
D-GIN cons.	0	6	0.434 ± 0.029	0.911 ± 0.011	dg601 cons.
D-GIN cons.	0	4	0.439 ± 0.026	0.909 ± 0.011	dg401 cons.
D-GIN cons.	6	7	0.461 ± 0.027	0.900 ± 0.012	dg761 cons.
D-GIN cons.	0	8	0.452 ± 0.030	0.904 ± 0.014	dg801 cons.
D-GIN cons.	6	4	0.440 ± 0.030	0.909 ± 0.012	dg461 cons.
D-GIN cons.	6	6	0.442 ± 0.026	0.908 ± 0.011	dg661 cons.

**Table A17:** Shows the logP RMSE and $r^{2}$ results for the D-MPNN model type used during this survey. The last column consists of the unique identifier. Training strategy 0 represents a training strategy combining logD, logS, and logP, 1 represents a strategy combining logD and logP, 2 stands for a combination of logD and logS, 3 represents a strategy using logS and logP, 4 represents a strategy using logD, 5 represents a strategy using logS, and 6 represents a strategy using logP.

Model	Training	Featurization	logD	$r^{2}$	Unique
Type	Strategy	Strategy	RMSE		ID
D-MPNN	6	6	0.540 ± 0.019	0.863 ± 0.012	dmp661
D-MPNN	6	5	0.735 ± 0.026	0.746 ± 0.019	dmp561
D-MPNN	0	8	0.583 ± 0.020	0.845 ± 0.012	dmp802
D-MPNN	0	6	0.551 ± 0.022	0.861 ± 0.012	dmp601
D-MPNN	6	4	0.556 ± 0.019	0.856 ± 0.011	dmp461
D-MPNN	0	5	0.717 ± 0.027	0.758 ± 0.021	dmp501
D-MPNN	6	3	0.570 ± 0.019	0.847 ± 0.012	dmp362
D-MPNN	6	7	0.629 ± 0.023	0.815 ± 0.014	dmp762
D-MPNN	0	7	0.605 ± 0.020	0.828 ± 0.012	dmp702
D-MPNN	6	8	0.593 ± 0.021	0.834 ± 0.014	dmp862
D-MPNN	0	4	0.571 ± 0.022	0.851 ± 0.012	dmp401
D-MPNN	0	3	0.552 ± 0.018	0.857 ± 0.011	dmp301
D-MPNN cons.	6	5	0.533 ± 0.031	0.866 ± 0.015	dmp562 cons.
D-MPNN cons.	0	6	0.444 ± 0.024	0.907 ± 0.010	dmp601 cons.
D-MPNN cons.	6	6	0.452 ± 0.026	0.904 ± 0.011	dmp661 cons.
D-MPNN cons.	0	5	0.524 ± 0.031	0.870 ± 0.015	dmp501 cons.
D-MPNN cons.	0	3	0.461 ± 0.027	0.900 ± 0.011	dmp301 cons.
D-MPNN cons.	0	4	0.463 ± 0.027	0.899 ± 0.011	dmp401 cons.
D-MPNN cons.	6	3	0.464 ± 0.026	0.899 ± 0.012	dmp362 cons.
D-MPNN cons.	6	8	0.471 ± 0.028	0.895 ± 0.012	dmp862 cons.
D-MPNN cons.	0	8	0.475 ± 0.027	0.894 ± 0.011	dmp802 cons.
D-MPNN cons.	0	7	0.484 ± 0.028	0.890 ± 0.012	dmp701 cons.
D-MPNN cons.	6	4	0.454 ± 0.026	0.903 ± 0.012	dmp462 cons.
D-MPNN cons.	6	7	0.488 ± 0.027	0.888 ± 0.012	dmp761 cons.

**Table A18:** Shows the logP RMSE and $r^{2}$ results for the GIN model type used during this survey. The last column consists of the unique identifier. Training strategy 0 represents a training strategy combining logD, logS, and logP, 1 represents a strategy combining logD and logP, 2 stands for a combination of logD and logS, 3 represents a strategy using logS and logP, 4 represents a strategy using logD, 5 represents a strategy using logS, and 6 represents a strategy using logP.

Model	Training	Featurization	logD	$r^{2}$	Unique
Type	Strategy	Strategy	RMSE		ID
GIN	0	3	0.752 ± 0.040	0.742 ± 0.025	g302
GIN	0	8	0.750 ± 0.029	0.738 ± 0.018	g802
GIN	0	4	0.716 ± 0.032	0.758 ± 0.019	g402
GIN	0	5	0.902 ± 0.033	0.624 ± 0.026	g502
GIN	6	5	0.894 ± 0.036	0.626 ± 0.026	g561
GIN	0	7	0.717 ± 0.033	0.758 ± 0.019	g701
GIN	6	7	0.727 ± 0.032	0.753 ± 0.022	g761
GIN	6	3	0.739 ± 0.030	0.744 ± 0.019	g361
GIN	6	8	0.741 ± 0.027	0.743 ± 0.018	g862
GIN	6	6	0.871 ± 0.033	0.643 ± 0.024	g662
GIN	6	4	0.725 ± 0.037	0.753 ± 0.025	g462
GIN	0	6	0.883 ± 0.036	0.640 ± 0.026	g601
GINcons.	6	7	0.537 ± 0.035	0.864 ± 0.016	g761 cons.
GINcons.	6	8	0.548 ± 0.034	0.858 ± 0.016	g861 cons.
GINcons.	0	4	0.539 ± 0.035	0.863 ± 0.015	g402 cons.
GINcons.	0	7	0.539 ± 0.034	0.863 ± 0.015	g701 cons.
GINcons.	6	4	0.534 ± 0.039	0.865 ± 0.018	g462 cons.
GINcons.	0	6	0.612 ± 0.038	0.823 ± 0.019	g601 cons.
GINcons.	6	6	0.606 ± 0.037	0.827 ± 0.019	g662 cons.
GINcons.	6	3	0.544 ± 0.035	0.860 ± 0.015	g362 cons.
GINcons.	0	3	0.547 ± 0.036	0.859 ± 0.015	g302 cons.
GINcons.	0	8	0.555 ± 0.033	0.855 ± 0.014	g802 cons.
GINcons.	6	5	0.614 ± 0.038	0.822 ± 0.019	g561 cons.
GINcons.	0	5	0.618 ± 0.037	0.820 ± 0.018	g502 cons.

**Table A19:** Shows the logP RMSE and $r^{2}$ results for the non-GNN model types used during this survey. The last column consists of the unique identifier. Training strategy 0 represents a training strategy combining logD, logS, and logP, 1 represents a strategy combining logD and logP, 2 stands for a combination of logD and logS, 3 represents a strategy using logS and logP, 4 represents a strategy using logD, 5 represents a strategy using logS, and 6 represents a strategy using logP.

Model	Training	Featurization	logD	$r^{2}$	Unique
Type	Strategy	Strategy	RMSE		ID
KNN	logP	13	0.939 ± 0.029	0.586 ± 0.024	KNN13
KNN	logP	14	0.995 ± 0.029	0.534 ± 0.025	KNN14
KNN	logP	15	1.065 ± 0.032	0.467 ± 0.027	KNN15
KNN	logP	10	1.082 ± 0.035	0.450 ± 0.023	KNN10
KNN	logP	12	1.087 ± 0.033	0.445 ± 0.023	KNN12
KNN	logP	16	1.100 ± 0.034	0.431 ± 0.026	KNN16
KNN	logP	11	1.103 ± 0.035	0.428 ± 0.026	KNN11
KNN	logP	17	0.744 ± 0.019	0.740 ± 0.014	KNN17
RF	logP	13	0.814 ± 0.029	0.688 ± 0.023	RF13
RF	logP	15	0.479 ± 0.022	0.892 ± 0.010	RF15
RF	logP	17	0.472 ± 0.023	0.895 ± 0.010	RF17
RF	logP	14	0.472 ± 0.024	0.895 ± 0.011	RF14
RF	logP	12	0.893 ± 0.030	0.625 ± 0.021	RF12
RF	logP	16	0.470 ± 0.024	0.896 ± 0.011	RF16
RF	logP	10	0.921 ± 0.031	0.601 ± 0.022	RF10
RF	logP	11	0.928 ± 0.029	0.595 ± 0.022	RF11
SVM	logP	14	0.572 ± 0.021	0.846 ± 0.012	SVM14
SVM	logP	12	0.809 ± 0.027	0.692 ± 0.018	SVM12
SVM	logP	16	0.628 ± 0.020	0.815 ± 0.012	SVM16
SVM	logP	17	0.493 ± 0.030	0.886 ± 0.013	SVM17
SVM	logP	10	0.833 ± 0.029	0.673 ± 0.020	SVM10
SVM	logP	11	0.827 ± 0.028	0.678 ± 0.020	SVM11
SVM	logP	13	0.782 ± 0.029	0.713 ± 0.020	SVM13
SVM	logP	15	0.602 ± 0.021	0.830 ± 0.013	SVM15
